# A_2__*B*_ Adenosine Receptors and Sphingosine 1-Phosphate Signaling Cross-Talk in Oligodendrogliogenesis

**DOI:** 10.3389/fnins.2021.677988

**Published:** 2021-05-26

**Authors:** Elisabetta Coppi, Francesca Cencetti, Federica Cherchi, Martina Venturini, Chiara Donati, Paola Bruni, Felicita Pedata, Anna Maria Pugliese

**Affiliations:** ^1^Department of Neuroscience, Psychology, Drug Research and Child Health (NEUROFARBA), Section of Pharmacology and Toxicology, University of Florence, Florence, Italy; ^2^Department of Experimental and Clinical Biomedical Sciences, University of Florence, Florence, Italy

**Keywords:** adenosine, sphingosine kinase (SphK), remyelination, K+ channels, oligodendrocyte differentiation, sphingosine-1-phosphate, oligodendrocyte progenitor cells (OPCs), A_2B_ receptors

## Abstract

Oligodendrocyte-formed myelin sheaths allow fast synaptic transmission in the brain. Impairments in the process of myelination, or demyelinating insults, might cause chronic diseases such as multiple sclerosis (MS). Under physiological conditions, remyelination is an ongoing process throughout adult life consisting in the differentiation of oligodendrocyte progenitor cells (OPCs) into mature oligodendrocytes (OLs). During pathological events, this process fails due to unfavorable environment. Adenosine and sphingosine kinase/sphingosine 1-phosphate signaling axes (SphK/S1P) play important roles in remyelination processes. Remarkably, fingolimod (FTY720), a sphingosine analog recently approved for MS treatment, plays important roles in OPC maturation. We recently demonstrated that the selective stimulation of A_2__*B*_ adenosine receptors (A_2__*B*_Rs) inhibit OPC differentiation *in vitro* and reduce voltage-dependent outward K^+^ currents (I_*K*_) necessary to OPC maturation, whereas specific SphK1 or SphK2 inhibition exerts the opposite effect. During OPC differentiation A_2__*B*_R expression increases, this effect being prevented by SphK1/2 blockade. Furthermore, selective silencing of A_2__*B*_R in OPC cultures prompts maturation and, intriguingly, enhances the expression of S1P lyase, the enzyme responsible for irreversible S1P catabolism. Finally, the existence of an interplay between SphK1/S1P pathway and A_2__*B*_Rs in OPCs was confirmed since acute stimulation of A_2__*B*_Rs activates SphK1 by increasing its phosphorylation. Here the role of A_2__*B*_R and SphK/S1P signaling during oligodendrogenesis is reviewed in detail, with the purpose to shed new light on the interaction between A_2__*B*_Rs and S1P signaling, as eventual innovative targets for the treatment of demyelinating disorders.

## Introduction

In the central nervous system (CNS), oligodendrocytes (OLs) are responsible for myelin production, which allows fast signal transmission and provides metabolic support to axons (Nave, 2010; Saab et al., 2016). During development, OLs are generated in the germinal zones of the brain, i.e., the subventricular zone (SVZ) (Yu et al., 1994), from migratory bipolar oligodendrocyte precursor cells (OPCs), which are renowned for the expression of the proteoglycan nerve-glial antigen 2 (NG2) (Grinspan, 2002; Brazel et al., 2004). Thanks to their migratory ability, OPCs spread and populate the embryonic brain and spinal cord (Emery, 2010) to differentiate into myelinating OLs. However, a pool of immature OPCs, comprising the 5-8% of total glial cells (Levine et al., 2001), persists within the adult CNS where they represent the major population of cycling cells (Dawson et al., 2003). This process guarantees, under physiological conditions, myelin turnover and remodeling in response to life experience (Malerba et al., 2015; Bergles and Richardson, 2016) and, in conditions of tissue damage, the generation of new OLs able to remyelinate the brain after a lesion or injury, an ability that might be lost through normal aging or chronic diseases. However, under pathological conditions such those characterized by chronic neuroinflammation and neurodegeneration, this process fails, leading to improper nerve conduction due to discontinuities in the myelin sheet (Nave, 2010; Franklin and Ffrench-Constant, 2017). For this reason, it is of critical relevance to identify innovative targets able to encourage OPC differentiation toward the mature, myelin-producing OL phenotype.

Among the factors influencing oligodendrogliogenesis, interest has been focused in the last years on the neuromodulator adenosine (Coppi et al., 2021), on one hand, and on the pleiotropic signaling molecule sphingosine 1-phosphate (S1P).

This review will focus on S1P and adenosine receptor-mediated effects in oligodendrocyte progenitors relevant for oligodendrogenesis and their possible functional interplay with the purpose to deepen the knowledge of molecular mechanisms involved in A_2__*B*_ receptor- (A_2__*B*_R-) or S1P-mediated effects and their cross-talk in oligodendrocyte biology.

### Oligodendrocyte Differentiation

Before being able to produce myelin, oligodendroglial cells progress through a series of highly regulated steps of differentiation from OPCs to mature OLs (de Castro and Bribian, 2005; Barateiro and Fernandes, 2014). This process is characterized by the loss of proliferative and migratory activity and the acquisition of an elaborate and highly ramified morphology (de Castro and Bribian, 2005). Oligodendrogliogenesis implicates a sequence of distinct maturational stages each of them identified by distinct morphological changes and by the expression of specific antigens (Levi et al., 1986; Gard and Pfeiffer, 1990; Warrington et al., 1992; Jung et al., 1996). On these bases, three major stages of differentiation have been proposed: a proliferating, bipolar OPC phase, characterized by the expression of platelet-derived growth factor (PDGF) receptor alpha (PDGFα), NG2 and the transcription factor Olig2 (Pringle et al., 1992; Ligon et al., 2006), a post-mitotic, moderately ramified pre-oligodendrocyte phase (pre-OL), positive for the markers O4 (Szuchet et al., 2011) and the recently deorphanized (Ciana et al., 2006) P2Y-like GPR17 receptor (Lecca et al., 2008; Emery, 2010; Fumagalli et al., 2011; Coppi et al., 2013b), and a mature myelinating OL phase, characterized by a highly ramified morphology and by the expression of galactocerebroside (GC), a specific marker for the oligodendrocyte membrane, myelin specific structural proteins such as 2’,3’-Cyclic-nucleotide-3’-phosphodiesterase (CNPase), myelin associated glycoprotein (MAG) and myelin basic protein (MBP) (Scolding et al., 1989; Zhang, 2001; Szuchet et al., 2011; Coppi et al., 2013a, 2015). Mature OLs synthesize large amounts of myelin, giving rise to multilamellar myelin sheaths that wrap and insulate neuronal axons which allow electrical isolation and saltatory conduction of electric impulses.

It is known that, during their maturation, oligodendroglial cells display functional voltage-gated ion channels whose expression changes during differentiation (Sontheimer et al., 1989; Barres et al., 1990; Williamson et al., 1997; Spitzer et al., 2019) including either inward or outward rectifying K^+^ channels (Kir and Kv, respectively), Na^+^ channels (Nav) and different subtypes of Ca^2+^ channels (Cav) (Verkhratsky et al., 1990).

During mouse brain development, the first ion channel subtype detected in OPCs is the Kv voltage-dependent, outwardly rectifying K^+^ conductance, at embryonic day 18 (E18) (Spitzer et al., 2019). However, a fraction of OPCs are described to express also Na_*V*_ currents that sharply increase around birth. This OPC population, with high Na_*V*_ and K_*V*_ densities, reflect a proliferative and migratory state during myelin generation (Gautier et al., 2015; Spitzer et al., 2019). Intriguingly, not all OPCs express Na_*V*_, but only a fraction, described to be around 60% (Kettenmann et al., 1991). Of note, a subpopulation of electrically excitable, spiking, NG2^+^ OPCs, able to generate full action potentials when stimulated by depolarizing current injection, have been described in brain slices (Karadottir et al., 2008), but the functional role of this “electrically excitable” OPC subpopulation is still unknown. Of note, single action potentials have also been detected in a minority of cultured OPCs (Barres et al., 1990).

The functional role of action potentials (APs) in OPC is still a matter of debate since, up to now, no functional differences have been detected between firing and not-firing OPCs (Bonetto et al., 2020). As long as OPCs undergo functional maturation, the density of Nav currents decreases as well as the expression of outward Kv conductances. In this phase, OPC differentiation potential declines and thus it can be considered a “quiescent” OPC state.

Among Kv currents, OPCs show outward conductances mainly composed by tetraetylammonium (TEA)-sensitive, delayed rectifying K^+^ currents (I_*K*_) (Sontheimer and Kettenmann, 1988) characterized by scarce time- and voltage-dependent inactivation and by a threshold for activation around -40 mV (Gutman et al., 2005). They also express a transient outward K^+^ current, or I_*A*_ (Gallo et al., 1996; Coppi et al., 2013b; Coppi et al., 2015), characterized by a rapid time-dependent inactivation (approximately 50 ms) and a voltage-dependent inactivation at potentials above -80 mV.

During maturation, outward K^+^ conductances (both I_*K*_ and I_*A*_), as well as I_*Na*_, undergo a strong downregulation up to almost completely disappearance in mature OLs (Sontheimer and Kettenmann, 1988; Sontheimer et al., 1989; Barres et al., 1990; Coppi et al., 2013a). Concomitantly, a gradual increase in the expression of inwardly rectifying K^+^ currents (Kir), activated at potentials lower than −100 mV, appears. Indeed, Kir currents are the main conductance observed in mature OLs (Knutson et al., 1997). Among the mentioned currents, TEA-sensitive I_*K*_ are crucially linked to cell cycle regulation and hence to myelin formation (Chittajallu et al., 2005), as demonstrated by the fact that when OPC cultures are grown in the presence of TEA, a significant inhibition of their proliferation and differentiation is observed (Gallo et al., 1996; Knutson et al., 1997; Chittajallu et al., 2005; Coppi et al., 2013b). Hence, compounds that modulate these currents may affect oligodendrocyte proliferation and myelination, as well as neurotransmitters, cytokines and growth factors acting on specific metabotropic receptors described to modify K^+^ current expression in OPCs (Stellwagen and Malenka, 2006; Zonouzi et al., 2011; Lundgaard et al., 2013; Malerba et al., 2015; Spitzer et al., 2019).

### Adenosine as a Neuromodulator

Adenosine is an intermediary metabolite acting as a building molecule for nucleic acids and a component of the biological energy currency ATP. In addition, adenosine is one of the most evolutionarily ancient signaling molecules (Verkhratsky and Burnstock, 2014), acting through the stimulation of four distinct adenosine-sensitive metabotropic P1 receptors denoted as A_1_, A_2__*A*_, A_2__*B*_ and A_3_ adenosine receptors (A_1_Rs, A_2__*A*_Rs, A_2__*B*_Rs and A_3_Rs) (Fredholm et al., 2011). These receptors are widely expressed both in the periphery or in the central nervous system (CNS) and have been implicated in a myriad of biological functions (Pedata et al., 2007; Coppi et al., 2012).

High extracellular concentrations of the nucleoside adenosine are found under conditions of tissue damage or when an imbalance in oxygen supply occurs (Latini et al., 1999; Pedata et al., 2014). Adenosine is short-lived in the extracellular space due to enzymatic degradation by adenosine deaminase (ADA) or adenosine kinase (AK) (Chen et al., 2013) or re-uptake operated by the equilibrative nucleoside transporters (ENT) isoforms ENT1 and ENT2 (Inoue, 2017), so its effects in the CNS are mainly described as autocrine and/or paracrine.

Among the four different P1 receptor subtypes, different affinities have been shown for the endogenous ligand. The activation of A_1_Rs is achieved as long as extracellular adenosine falls in the low nanomolar range (1-10 nM), a concentration generally present in most tissues and organs throughout the body (Latini and Pedata, 2001). A_1_R-mediated signal activates G_*i/o*_ proteins leading to the inhibition of adenylyl cyclase (AC) and to a decrease in intracellular cAMP levels (Antonioli et al., 2019). A_1_Rs are the predominant P1 receptor subtype in the CNS, with high levels of expression reported in the cerebral cortex, hippocampus, cerebellum, thalamus, brainstem and spinal cord. It is well known that A_1_R activation inhibits synaptic transmission (Corradetti et al., 1984; Dunwiddie, 1984) leading to sedative, anticonvulsant (Muzzi et al., 2013) and anxiolytic (Vincenzi et al., 2016) effects in the CNS whereas, at cardiovascular levels, they are potent bradycardic agents (Jacobson and Gao, 2006).

The A_2__*A*_R subtype is known to stimulate AC by G_*s*_ protein activation, leading to the production of cAMP which acts as a second messenger by activating protein kinase A (PKA) (Antonioli et al., 2019). Within the brain, this receptor subtype is widely expressed with particularly high levels found in the striatum/caudate-putamen nuclei (Peterfreund et al., 1996). In the periphery, its expression is abundant on blood vessels and inflammatory/immune cells (Yu L. Q. et al., 2004). The functional effect of A_2__*A*_Rs in the brain is at variance from A_1_R subtype, as they enhance glutamate release and promote cell excitability (Goncalves and Ribeiro, 1996; Lopes et al., 2002). In accordance, A_2__*A*_R activation participates to excitotoxic damage due to extracellular glutamate overload during an ischemic-like insult obtained *in vitro* by oxygen and glucose deprivation (OGD) (Colotta et al., 2012; Maraula et al., 2013; Maraula et al., 2014). Concerning peripheral functions of A_2__*A*_Rs, it is worth to note that adenosine, thanks to its actions on this receptor subtype, is one of the most powerful endogenous anti-inflammatory agents (Antonioli et al., 2019). Indeed, A_2__*A*_Rs are highly expressed in inflammatory cells including lymphocytes, granulocytes and monocytes/macrophages, where their activation reduces pro-inflammatory cytokines, i.e., tumor necrosis factor-alpha (TNFα), interleukin-1β (IL-1 β) and interleukin-6 (IL-6) (Varani et al., 2011) and enhances the release of anti-inflammatory mediators, such as interleukin-10 (IL-10) (Bortoluzzi et al., 2016).

The A_2__*B*_R subtype is somewhat the most enigmatic and less studied among the four P1 receptors as its pharmacological and physiological characterization has long been precluded by the lack of suitable ligands able to discriminate among the other adenosine receptor subtypes (Popoli and Pepponi, 2012; Chandrasekaran et al., 2019). The central distribution of A_2__*B*_Rs on neurons and glia is scarce but widespread, whereas in the periphery abundant levels of A_2__*B*_Rs are observed in bronchial epithelia, smooth muscles, inflammatory cells such as mast, neutrophils and monocytes, vasculature and digestive tracts like ileum and colon (Feoktistov et al., 1998; Chandrasekaran et al., 2019). It is reported that A_2__*B*_R activation stimulates G_*s*_ and, in some cases, also G_*q/*__11_ proteins, thus activating either or both cAMP-pathway and IP_3_- related pathways. In this second case, the activation of phospholipase C (PLC) leads to the production of inositol-(1,4,5)-trisphosphate (IP_3_) and diacylglycerol (DAG), that increases the intracellular levels of Ca^2+^ and activates protein kinase C (PKC), respectively (Antonioli et al., 2019).

Differently from high affinity A_1_Rs and A_2__*A*_Rs, activated by physiological levels of extracellular adenosine (low nM and high nM, respectively (Coppi et al., 2020b), the A_2__*B*_R needs much higher adenosine concentrations (in the μM range) reached only in conditions of tissue damage or injury (Chandrasekaran et al., 2019). Such a low affinity of A_2__*B*_Rs for the endogenous agonist implies that they represent a good therapeutic target, since they are activated only by high adenosine efflux reached under pathological conditions or injury, when a massive release of adenosine occurs (Popoli and Pepponi, 2012) or that they can be driven to function by selective agonists (Coppi et al., 2020b). All the adenosine receptors mentioned above are also associated with mitogen-activated protein kinase (MAPK) pathways, which involves the activation of extracellular signal-regulated kinase 1 and 2 (ERK1 and ERK2), whose action can mediate numerous cellular responses, and of JUN N-terminal kinase (JNK) and p38 MAPK (Melani et al., 2009). In particular, A_1_Rs and A_2__*B*_Rs promote the activation of ERK1, ERK2, p38 MPAK and JNK, instead A_2__*A*_Rs and A_3_Rs are involved only in the stimulation of ERK1 and ERK2 signaling (Antonioli et al., 2013).

As mentioned above for the cognate A_2__*A*_R subtype, A_2__*B*_R activation within the CNS is reported to increase glutamate release (Goncalves et al., 2015; Fusco et al., 2019) but the mechanism is at variance from the former. In fact, Cunha and co-workers demonstrated that the A_2__*B*_R selective agonist BAY60-6583 attenuates the predominant A_1_R-mediated inhibitory control of synaptic transmission in the CA1 hippocampus (Goncalves et al., 2015). These data are consistent with the relatively abundant expression of A_2__*B*_Rs in hippocampal presynaptic sites, demonstrated by means of synaptosome preparation, reported by the same authors (Goncalves et al., 2015). The facilitatory effect of A_2__*B*_Rs on glutamatergic neurotransmission was confirmed by us in acute hippocampal slices by using the electrophysiological protocol of paired pulse facilitation (PPF) (Fusco et al., 2019). We reported that A_2__*B*_R activation decreases PPF in the CA1 hippocampus, an effect known to be ascribed to enhanced glutamate release (Zucker and Regehr, 2002). Furthermore, the effect of BAY60-6583 was prevented not only by the A_2__*B*_R antagonists MRS1754 and PSB-603, but also by the A_1_R blocker DPCPX (Fusco et al., 2019), confirming the fact that A_1_R activation in necessary for the enhancing effect of A_2__*B*_Rs on glutamate release. Furthermore, we extended results to a newly synthetized A_2__*B*_R-selective agonist, the recently described BAY60-6583-analog P453 (Betti et al., 2018) which proved higher affinity for the A_2__*B*_R (50 nM *vs*. 200 nM, respectively) than the commercially available BAY 60-6583. In addition, as reported for A_2__*A*_Rs, also A_2__*B*_R activation participates to OGD-induced synaptic failure in the hippocampus (Fusco et al., 2018).

In the periphery, A_2__*B*_Rs are present on hematic cells, such as lymphocytes and neutrophils, with the highest expression levels found on macrophages (Gessi et al., 2005; Yang et al., 2006). Here, A_2__*B*_Rs in most cases are co-expressed with A_2__*A*_Rs and their activation exert anti-inflammatory effects by inhibiting migration and vascular adhesion (Yang et al., 2006) of inflammatory cells (Wakai et al., 2001; Eckle et al., 2008).

The A_3_R subtype is known to couple to G_*i/o*_ proteins and to inhibit AC but, under particular conditions or in different cell types, activation of G_*q/*__11_ by A_3_R agonists has also been reported (Antonioli et al., 2019). This receptor subtype presents large interspecies differences, with only 74% sequence homology between rat and human (Koscso et al., 2011). Its expression is not uniform throughout the body: low levels are found in the brain and spinal cord whereas a predominance of this receptor subtype is described in peculiar regions at the periphery, i.e., in the testis, lung, kidneys, placenta, heart, brain, spleen and liver (Gessiaa et al., 2008). Interestingly, most of the cell types of the immune system express functional A_3_Rs on their surface (Hasko et al., 2008) and its activation is one of the most powerful stimuli for mast cells degranulation (Ramkumar et al., 1993).

### Adenosine in Oligodendrogliogenesis

Oligodendrocyte turnover is rather slow under physiological conditions and guarantees myelin turnover and remodeling in response to life experience (Bergles and Richardson, 2016). However, a disruption in this process, for example in case of a maturation block, could have devastating consequences during aging and in pathological conditions, such as multiple sclerosis (MS).

Recruitment of OPCs to injured areas is in fact one of the most important events to promote remyelination after CNS injury (Simon et al., 2011; Neumann et al., 2019). Unfortunately, it is still not clear why this process fails, or is insufficient to provide myelin repair, during chronic demyelinating diseases (Romanelli et al., 2016). In fact, most OPCs fail to mature into myelin-producing OLs in MS, indicating that remyelination by adult OPCs is hindered principally due to a failure of OPC differentiation into mature OLs rather than a failure of repopulation or migration of OPCs (Chang et al., 2002; Patel and Klein, 2011).

A deep knowledge of the intricate processes regulating OPC maturation to OLs is mandatory to investigate new therapeutic targets aimed at counteracting demyelinating diseases and repair myelin damage in the long run.

Among the neuromodulators contributing to the balance between proliferating, immature OPCs and myelinating, mature OLs there are purines (Stevens et al., 2002; Fields, 2004; Fields and Burnstock, 2006) and, in particular, adenosine (Coppi et al., 2020b).

All P1 receptors are expressed by maturating oligodendroglial progenitors as well as by mature OLs (Stevens et al., 2002; Coppi et al., 2020a) and exert a key role in cell development (Coppi et al., 2015, 2020b). Furthermore, the expression by oligodendrocytes of the nucleoside transporters ENT1 and ENT2, as well as adenosine degrading enzymes ADA and AK, suggests that these cells are able to sense and finely tune extracellular adenosine levels (Gonzalez-Fernandez et al., 2014), thus supporting the notion that purinergic signaling exerts a prominent role in these cells (Burnstock et al., 2011).

**FIGURE 1:**
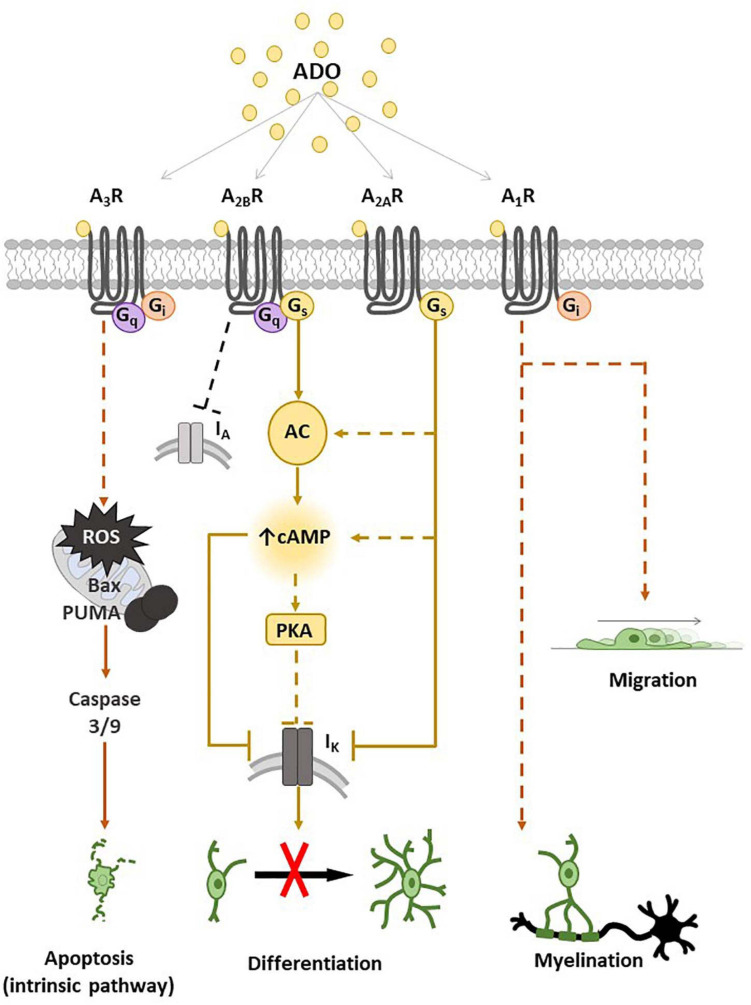
Adenosine receptor expression and main transduction pathways activated in oligodendrocyte progenitor cells (OPCs) and oligodendrocytes (OLs). Schematic representation of A_1_, A_2__*A*_, A_2__*B*_ and A_3_ receptor (A_1_R, A_2__*A*_R, A_2__*B*_R and A_3_R) effects on OPCs and main intracellular pathways involved. The activation of A_1_R by adenosine (ADO) or other selective receptor agonists promotes myelination and migration. The stimulation of G_*s*_-coupled A_2__*A*_R and/or A_2__*B*_R leads to adenylyl cyclase (AC) activation with a consequent increase in intracellular cyclic adenosine monophosphate (cAMP), which closes I_*K*_ channels and inhibits OPC differentiation, probably by a mechanism involving protein kinase A (PKA). A_3_R stimulation induces OPC apoptosis by the activation of an intrinsic pathway, i.e., through reactive oxygen species (ROS) production and activation of Bcl-2-associated X (Bax), p53-upregulated modulator of apoptosis (PUMA) and caspase 3/9. Dotted lines are used when the intracellular pathway/s have not been described.

Indeed, our research group contributed to demonstrate that adenosine can affect numerous OPC functions such as migration, proliferation and maturation (Fields and Stevens-Graham, 2002; Stevens et al., 2002; Coppi et al., 2013a,b, 2015; Cherchi et al., 2021), with distinct effects mediated by different receptor subtypes, as summarized in Figure 1.

It has been demonstrated that tonic electrical stimulation of co-cultures of OPCs with dorsal root ganglion neurons induces the release of adenosine that inhibits OPC proliferation and promotes their maturation toward pre-myelinating OLs, an effect blocked by a cocktail of A_1_R, A_2__*A*_R and A_3_R antagonists (Stevens et al., 2002), suggesting that endogenous adenosine released in response to impulse activity promotes oligodendrocyte development and myelination. Furthermore, A_1_R agonists have been reported to stimulate OPC migration (Figure 1; Othman et al., 2003).

Concerning the A_2__*A*_R subtype, our group of research demonstrated that the selective A_2__*A*_R agonist CGS21680 inhibits TEA- sensitive I_*K*_ currents in cultured OPCs and delays *in vitro* cell differentiation without affecting neither cell viability nor proliferation (Coppi et al., 2013a). These effects were completely prevented in the presence of the selective A_2__*A*_R antagonist SCH58261 (Coppi et al., 2013a). In keeping with data demonstrating that TEA impairs OPC maturation (Attali et al., 1997; Gallo et al., 1996; Coppi et al., 2013b) and blocks myelin deposition in the embryonic spinal cord (Shrager and Novakovic, 1995) and ovine OPCs (Soliven et al., 1988), it appears that the G_*s*_-coupled A_2__*A*_R inhibits OPC differentiation by reducing I_*K*_ currents. In line with this assumption is the observation that selective activation of GPR17, a G_*i*_-coupled P2Y-like receptor, enhances TEA-sensitive I_*K*_ and improves OPC differentiation (Coppi et al., 2013b).

The less known adenosine receptor in OPCs is the A_3_R subtype. The only paper available in the literature demonstrates that the A_3_R agonist 2-CI-IB-MECA induces apoptosis of cultured oligodendroglial cells isolated from rat optic nerve (Gonzalez-Fernandez et al., 2014) and induces myelin loss in an *ex vivo* preparation of optic nerve, an effect prevented by the A_3_R antagonist MRS1220 (Gonzalez-Fernandez et al., 2014). Moreover MRS1220 also prevented optic nerve demyelination induced by *in vitro* ischemic-like conditions, i.e., OGD (Gonzalez-Fernandez et al., 2014). Thus, data suggest that adenosine, via activation of A_3_Rs, triggers oligodendrocyte death (Figure 1) and contributes to white matter ischemic damage.

### Role of A_2__*B*_Rs in OPCs and Oligodendrogliogenesis

Current research in the field of adenosine is ongoing thanks to the growing interest on this receptor subtype. The pharmacological and physiological characterization of A_2__*B*_Rs has long been precluded by the lack of suitable ligands (Popoli and Pepponi, 2012).

Very few data are available to date about the role of A_2__*B*_Rs in oligodendroglial cells, as this adenosine receptor subtype is somewhat the most enigmatic and less studied among the four P1 receptors. We recently found that A_2__*B*_Rs are crucially involved in OPC maturation by demonstrating that the selective A_2__*B*_R agonist BAY60-6583, and its recently synthetized analog P453 (Betti et al., 2018), inhibited *in vitro* OPC differentiation, as demonstrated by the reduced expression of myelin-related proteins such as MBP or MAG in primary purified OPC cultures (Coppi et al., 2020a). We also demonstrated that A_2__*B*_R activation reversibly inhibits TEA- sensitive, sustained I_*K*_, and 4-amynopyridine- (4-AP) sensitive, transient I_*A*_, conductances (Coppi et al., 2020a). As mentioned above, I_*K*_ are necessary to OPC maturation (Gallo et al., 1996), so this could be one of the mechanisms by which A_2__*B*_Rs inhibit myelin production. The AC activator forskolin mimicked BAY60-6583-mediated effect as it decreased I_*K*_ currents, in line with previous data (Soliven et al., 1988). Of note, a further application of BAY60-6583 in the presence of forskolin was devoid of effect, thus indicating that A_2__*B*_R activation inhibits I_*K*_ by increasing intracellular cAMP levels (Coppi et al., 2020a).

Data about an inhibitory role of A_2__*B*_R in myelin formation are consistent with recent findings from Manalo et al. (2020) who demonstrated that elevated cochlear adenosine levels in ADA^–/–^ mice is associated with sensorineural hearing loss (SNHL) due to cochlear nerve fiber demyelination and mild hair cell loss. Intriguingly, A_2__*B*_R-specific antagonists administered in ADA^–/–^ mice significantly restored auditory capacity, nerve fiber density and myelin compaction. The same authors also provided genetic evidence for A_2__*B*_R upregulation not only in ADA^–/–^ hear-impaired mice but also in age-related SNHL.

### Adenosine and Multiple Sclerosis

Multiple sclerosis is a chronic demyelinating disease of the CNS that leads to progressive neurological disability (Courtney et al., 2009). Immune system and inflammatory niche react against the myelin sheath that covers axon fibers altering neuronal transmission leading to the onset of a permanent condition associated with nerve decline. Since the extent and severity of nerve damage is heterogeneous, MS clinical symptoms may differ upon patients depending on the type and amount of the affected nerves. The treatments available for MS are almost limited aiming at reducing relapsing frequency or increasing the remitting speed of the disease with the major aim to manage symptoms.

Adenosine receptors are involved in inflammation and oligodendrogliogenesis, as reported above, and may represent potential therapeutic targets in the treatment of MS (Johnston et al., 2001; Stevens et al., 2002; Coppi et al., 2015; Cherchi et al., 2021). Compounds used in the treatment of MS, such as methotrexate and cladribine, have been shown to act as ligands at adenosine receptors, to exert their anti-inflammatory activities (Montesinos et al., 2000). Therefore, the beneficial actions of these drugs may also involve their ability to activate adenosine A_2_R and A_3_R in a compensatory manner to regulate cytokine expression (Johnston et al., 2001).

Experimental autoimmune encephalomyelitis (EAE) is the most frequently used animal model to study the immunopathogenesis of MS and to test the therapeutic efficacy of novel agents for preclinical study (Constantinescu et al., 2011). EAE can be induced by inoculating the animal with whole myelin or defined myelin protein, as myelin oligodendrocyte glycoprotein (MOG), with adjuvants, which lead to activation of autoreactive peripheral CD4 T-cells and their subsequent trafficking to the CNS by crossing the blood brain barrier (BBB).

The A_1_R is expressed on cells of the monocyte/macrophage lineage and a downregulation of A_1_R was found in both blood and brain from MS patients (Mayne et al., 1999; Johnston et al., 2001). Accordingly, activation of A_1_R is reported to protect from EAE damage (Chen et al., 2010; Wang et al., 2014; Liu G. P. et al., 2018) and A_1_R knockout (A_1_R^–/–^) exacerbates MOG-induced EAE pathology by increasing demyelination, axonal injury and neuroinflammation (Tsutsui et al., 2004). Finally, A_1_R activation improves myelin repair by recruiting OPCs in an experimental model of rat optic nerve demyelination (Asghari et al., 2013).

The role of A_2__*A*_Rs in MS is controversial. This receptor subtype is upregulated in human lymphocytes (Vincenzi et al., 2013) and in the CNS (Rissanen et al., 2013) of MS patients, thus suggesting an involvement in demyelinating pathologies. However, A_2__*A*_R overexpression does not correlate with different forms of the disease nor is affected by MS pharmacological treatments (Vincenzi et al., 2013). Surprisingly, A_2__*A*_R^–/–^ mice developed more severe EAE than wild type animals (Mills et al., 2012; Yao et al., 2012) and the adoptive transfer of peripheral blood cells lacking the A_2__*A*_R into wild type animals induced more severe EAE than both wild type and A_2__*A*_R^–/–^ mice (Mills et al., 2012). This suggests that A_2__*A*_R^–/–^ mice are susceptible to a severe acute form of EAE due to the lack of A_2__*A*_R-mediated anti-inflammatory effects. Indeed, in human lymphocytes, A_2__*A*_R agonist inhibits the release of proinflammatory cytokines, cell proliferation, the expression of the adhesion molecule VLA-4, and the activation of the transcription factor NF-κB; these effects were more evident in lymphocytes from MS patients in comparison to healthy subjects, in line with upregulation of A_2__*A*_Rs in MS lymphocytes (Vincenzi et al., 2013). Consistent with this, the activation of A_2__*A*_R signaling by selective agonist inhibits the EAE progression by suppressing the specific lymphocyte proliferation, reducing the infiltration of CD4^+^ T lymphocytes, increasing intracellular Ca^2^^+^ levels (Liu et al., 2016), and reducing the effects of Th1 stimulation on the BBB permeability (Liu Y. et al., 2018). Additional evidence demonstrates that A_2__*A*_R agonism in EAE leads to prevention of the disease when used in early disease stage (Ingwersen et al., 2016) whereas in late-stage EAE the number of foci with marked amount of myelin debris was higher in A_2__*A*_R^–/–^ mice than wild type (Ingwersen et al., 2016). These findings led to hypothesise that the relevance of A_2__*A*_R for the pathogenesis of chronic autoimmune neuroinflammation may depend on the time point or the compartment, i.e., the systemic immune response vs. the CNS, an issue that has been elegantly addressed by Rajasundaram in a recent review (Rajasundaram, 2018).

Little is known about the role of A_2__*B*_R in MS or EAE. Similarly to A_2__*A*_Rs, A_2__*B*_R expression increases in peripheral lymphoid tissues of EAE mice. However, up to now, evidences converge on indicating that A_2__*B*_R blockade is protective in EAE models. Indeed, Wei and colleagues demonstrated that A_2__*B*_R-specific antagonists or genetic ablation of the receptor attenuated the clinical signs of EAE and protect the CNS from immune damage, probably by eliminating adenosine-mediated IL-6 production (Wei et al., 2013). Furthermore, the A_2__*B*_R agonist BAY60-6583 reversed mesenchymal stem cells-induced downregulation of AQP4 expression in cultured astrocytes, that is protective for maintaining the integrity of BBB in EAE (Liu et al., 2020).

### Sphingosine 1-Phoshate and Its Metabolism

Sphingosine 1-phosphate (S1P) is a natural sphingolipid present in plasma and tissues (Ksiazek et al., 2015) that acts as a modulator of different physiological and pathological processes, such as angiogenesis, vascular stability and permeability, T- and B-cell trafficking, as well as tumorigenesis (Hla et al., 2008). Circulating S1P is mainly produced and released by erythrocytes together with platelets and endothelial cells; it is mainly transported in association with apolipoprotein M (apoM) in high-density lipoprotein (HDL), while a small fraction is transported bound to albumin. ApoM can be considered a S1P chaperone protein (Obinata et al., 2019), which carries the sphingolipid in blood and interstitial fluids, facilitates S1P signaling in tissues and is responsible for physio-pathological effects different from that elicited by albumin-bound S1P. ApoM-bound S1P is able to exert multiple effects such as reduction of vascular inflammation, improvement of endothelial barrier function, inhibition of oxidized low-density lipoprotein- (ox-LDL-) induced inflammation, modification of BBB permeability and protection from bacteria-induced inflammatory responses (Christoffersen et al., 2011; Galvani et al., 2015; Christensen et al., 2016; Zheng et al., 2019). Endothelial cells embracing the BBB are able to secrete S1P into the blood as well as into brain compartments (Hajny and Christoffersen, 2017). S1P concentration is much higher in blood and lymph at μM range than in interstitial tissues due to the activity of S1P degrading enzymes. This concentration gradient, termed the “vascular S1P gradient” causes lymphocyte and hematic cell trafficking from lymph organs or bone marrow to the circulation (Schwab et al., 2005; Pappu et al., 2007; Hla et al., 2008; Cyster and Schwab, 2012). Enhanced vascular permeability, which occurs especially during inflammation, induces a burst of S1P that becomes available in the extravascular milieu, suggesting that the vascular S1P gradient may contribute to physiological and pathological conditions.

**FIGURE 2:**
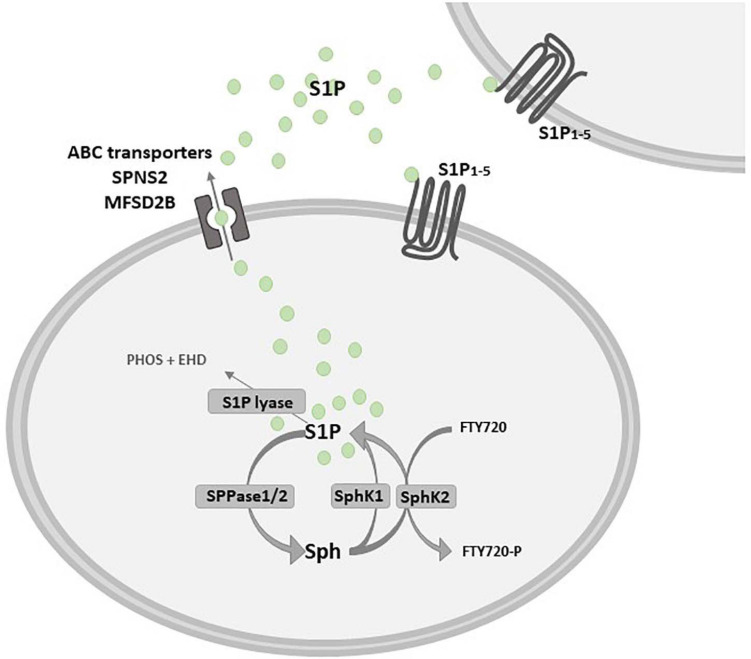
Schematic diagram describing the inside-out signaling of S1P. Sphingosine (Sph) kinase 1 and/or 2 (SphK1/SphK2) activation leads to sphingosine 1-phosphate (S1P) production that can act as an intracellular mediator or is extracellularly released to bind specific S1P receptors (S1P_1__–__5_). S1P release in the extracellular milieu depends on specific and/or unspecific transporters, such as the S1P transporter spinster homolog 2 (SPNS2) and the major facilitator superfamily transporter 2b (MFSD2B), as well as some ATP-binding cassette (ABC) family members, that are critically implicated in the so called S1P “inside-out signaling”. S1P is dephosphorylated by sphingosine 1-phosphate phosphatase 1 or 2 (SPPase1/2) or it is irreversibly degraded by S1P lyase in phosphoethanolamine (PHOS) and hexadecanal (EHD). Fingolimod (FTY720) is a structural analog of Sph that is rapidly phosphorylated by SphK2 in FTY720-P.

The catabolic pathway of plasma membrane-derived complex sphingolipids, mainly sphingomyelin, gives rise to different bioactive molecules, among others S1P. The first event is the sphingomyelinase-dependent hydrolysis of sphingomyelin, that leads to the production of the pro-apoptotic ceramide (Proia and Hla, 2015). Ceramidase deacylates ceramide to sphingosine, which in turn is converted into S1P by ATP-dependent phosphorylation catalyzed by two isoforms of the enzyme sphingosine kinase (SphK), namely SphK1 and 2 (Figure 2). These isozymes have been cloned and characterized, and their encoding genes are localized in different chromosomes, 17 and 19 respectively, known to produce multiple splicing variants (Imamura et al., 2001; Alemany et al., 2007). SphK1 and SphK2 differ for subcellular localization, showing partial overlapping but sometimes different biological functions (Liu et al., 2002; Pyne et al., 2016). In this regard, although knockout mouse models for either SphK1 or SphK2 have no gross phenotypic alterations, the double null mutation is lethal to embryos due to alterations in vasculogenesis and hemorrhages (Allende et al., 2004; Mizugishi et al., 2005; Michaud et al., 2006). SphK1 and SphK2 have structural homologies and share five conserved catalytic domains including an ATP-binding motif related to that of the diacylglycerol kinase family (Pitson et al., 2000; Liu et al., 2002). However, the sequence of SphK2 shows additional regions at the N-terminal and central proline-rich sequences which are absent in SphK1 (Igarashi et al., 2003; Gao et al., 2012). A nuclear localization sequence (NLS) and a nuclear exporting sequence (NES) are responsible for SphK2 major localization in the nucleus, since overexpression of SphK2 or SphK1 fused with NLS, but not wild-type SphK1, causes an inhibition of DNA synthesis (Igarashi et al., 2003). Indeed, it has been demonstrated that SphK2 has a role in epigenetic regulation of gene expression being involved in the suppression of histone deacetylase (HDAC) activity (Hait et al., 2009). Moreover, SphK2 is present in five different splicing variants that have been reported to localize into other different intracellular compartments such as the cytosol, mitochondria and the endoplasmic reticulum (ER) (Maceyka et al., 2005; Okada et al., 2005; Strub et al., 2011).

Intriguingly, SphK2 displays paradoxical effect on cell survival. Indeed, SphK2 shows a BH3 putative pro-apoptotic domain, although apoptosis is induced via SphK2-Bcl-xL-interaction after overexpression of the kinase (Liu et al., 2003). Moreover, knockdown of SphK2 also affects apoptosis induced by transforming growth factor beta (TGFβ) in C2C12 myoblasts (Cencetti et al., 2013). Alternately, SphK2 is significantly elevated in a broad range of human cancers, including bladder, melanoma, breast, neuroblastoma and leukemia (Neubauer et al., 2016; Bruno et al., 2020); in agreement to SphK2 expression in cancer, SphK2 down-regulation has been demonstrated to decrease the proliferation of cancer cells (Van Brocklyn et al., 2005; Hait et al., 2005), and SphK2-deficient xenografts show a significantly delayed growth (Weigert et al., 2009), pointing at a crucial role of the kinase in carcinogenesis.

Concerning SphK1, its up-regulation is tumorigenic whereas down-regulation results in anti-cancer effects (Sarkar et al., 2005; Taha et al., 2005). The localization of SphK1 is mainly in the cytosol, but upon activation by several stimuli the kinase is recruited at the plasma membrane (Johnson et al., 2002). Moreover, although both SphKs can phosphorylate the sphingosine analog, immunomodulatory drug FTY720 (Fingolimod), SphK2 appears to be more efficient in this activity than SphK1 (Billich et al., 2003; Paugh et al., 2003). Indeed, in mice lacking SphK2, but not SphK1, lymphopenia and lymphocyte retention into lymphoid organs induced by FTY720 are completely lost (Allende et al., 2004; Kharel et al., 2005).

The levels of S1P are tightly regulated by modulation of both anabolic and catabolic pathways. Breakdown of S1P to sphingosine is reversibly triggered by S1P phosphatases (SPP1 and 2), as well as unspecific lipid phosphate phosphatase (LPP), otherwise S1P is irreversibly degraded to hexadecenal and phosphoethanolamine by S1P lyase (Hannun and Obeid, 2008; Figure 2). *De novo* sphingolipids pathway alternatively accounts for the production of ceramide, which is the main hub of the sphingolipid metabolism (Hannun and Obeid, 2008): this anabolic pathway is initiated by serine palmitoyl transferase that catalyzes the condensation of serine with palmitoyl-CoA to produce 3-keto-dihydrosphingosine, which is consequently converted into dihydrosphingosine (Hanada, 2003). Ceramide synthases catalyze the acylation of dihydrosphingosine in dihydroceramide and finally dihydroceramide desaturase is involved in the final step of the *de novo* synthesis by producing ceramide; besides representing a substrate for sphingomyelin synthase giving rise to sphingomyelin and for other complex sphingolipid biosynthetic pathways, also *de novo* synthesized ceramide can fuel the activity of ceramidases and then S1P production. Ceramide is then transported to the Golgi complex, where it serves as substrate for production of complex sphingolipids (Gault et al., 2010).

### Sphingosine 1-Phosphate Signaling

Extracellular stimuli including growth factors and cytokines such as epidermal growth factor (EGF), PDGF, vascular endothelial growth factor (VEGF), insulin-like growth factor- 1 (IGF-1), TNFα and TGFβ can induce the activation of SphK1 that translocates from cytosol to the plasma membrane to produce S1P (Donati et al., 2007; Bernacchioni et al., 2012; Bernacchioni et al., 2021). The bioactive sphingolipid can act in an autocrine or paracrine manner after its release in the extracellular milieu (called “inside-out signaling”), depending on specific and/or unspecific transporters, such as the S1P transporter spinster homolog 2 (SPNS2) (Hisano et al., 2012; Spiegel et al., 2019), the major facilitator superfamily transporter 2b (MFSD2B) (Kobayashi et al., 2018) and some, unspecific, ATP-binding cassette (ABC) transporters (Mitra et al., 2006; Figure 2).

**FIGURE 3:**
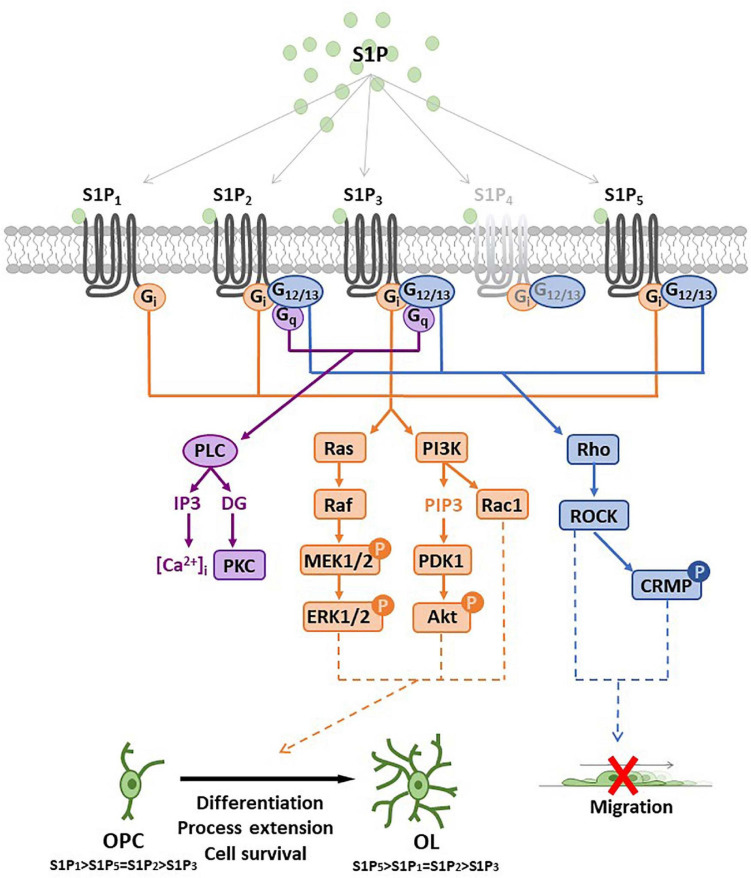
Sphingosine 1-phosphate receptor (S1PR) expression and main signal transduction pathways activated in oligodendrocyte progenitor cells (OPCs) and oligodendrocytes (OLs). Schematic representation of sphingosine 1-phosphate (S1P) G-protein coupled receptors (S1P_1__–__5_) and main signaling pathways involved after receptor activation. All S1P_1__–__5_ are coupled to G_*i*_ protein that activates Ras/PI3K pathways (in orange) promoting oligodendrocyte differentiation, process extension or cell survival. S1P_2__/__5_ are also coupled to G_12__/__13_ protein that activates Rho pathway (in blue) and reduces OPC migration or induces process retraction. S1P_2__/__3_ are coupled also with G_*q*_ protein that activates PLC leading to intracellular calcium increase and PKC activation. Abbreviation: mitogen-activated protein kinase (MEK); extracellular signal-regulated kinases (ERK); phosphatidylinositol 3-kinase (PI3K); phosphatidylinositol (3,4,5)-trisphosphate (PIP3); 3-phosphoinositide dependent protein kinase-1 (PDK1); protein kinase B (Akt); phospholipase C (PLC); protein kinase C (PKC); Rho-associated protein kinase 1 (ROCK); collapsin response mediator protein (CRMP); inositol triphosphate (IP3); diacylglycerol (DG).

The bioactive S1P selectively binds to high affinity cell surface G protein-coupled receptors (GPCRs; S1PRs), named S1P_1_, S1P_2_, S1P_3_, S1P_4_, and S1P_5_ (Ishii et al., 2004) that have been involved in the majority of physiological and pathological actions evoked by S1P such as immune response, cardiovascular functions, cancer, atherosclerosis (Cartier and Hla, 2019) as well as MS (Strub et al., 2010; Maceyka et al., 2012). While S1P_1_, S1P_2_ and S1P_3_ show broad tissue expression, S1P_4_ displays quite selective localization in immune system and S1P_5_ is primarily expressed in the spleen, on natural killer cells and other lymphocytes, and in CNS, mainly in oligodendrocytes (Mutoh et al., 2012). S1PRs exhibit a distinct capacity to couple to different G proteins thus activating different patterns of intracellular signaling cascades (Figure 3). S1P_1_ exclusively activates members of the G_*i*_ family, whereas S1P_2_ and S1P_3_ have a broader coupling profile and not only activate G_*i*_ but also G_*q*_ and G_12__/__13_. Moreover, activation of Rho, Rac and other small GTPases induces downstream signaling pathways including MAPK, phosphoinositide 3-kinase/Akt, Rho-associated protein kinase (ROCK). Further downstream effectors of S1PRs include AC, PLC, PKC and intracellularCa^2^^+^ transients (Windh et al., 1999; Maceyka et al., 2012; Figure 3). In addition, several lines of evidence show that S1P can also act as an intracellular messenger (Kohno et al., 2006; Olivera and Spiegel, 2001; Strub et al., 2010; Maceyka et al., 2012) regulating fundamental biological processes, such as gene expression, mitochondrial functions and inflammation by interacting with intracellular targets, including HDACs, E3 ubiquitin ligases and prohibitin 2. Indeed, it has been shown that SphK2 is associated with histone H3, and regulates histone tail acetylation via S1P-medated inhibition of HDAC, thus inducing chromatin remodeling and gene transcription (Hait et al., 2009). Interestingly, TNFα-induced activation of NF-κB requires SphK1 activity and S1P production. In particular, pro-survival TNF signaling requires TRADD-mediated recruitment of TNF receptor-associated factor 2 (TRAF2), which is the prototypical member of E3 ubiquitin ligase (Alvarez et al., 2010). Recruitment of TRAF2 to the TNFα-induced signaling complex results in the polyubiquitination of receptor interacting protein 1 (RIP1) preventing procaspase 8 cleavage that leads to apoptosis. Instead, polyubiquitinated RIP1 is capable of binding and activating IκB kinase (IKK), that phosphorylates the inhibitor of NF-κB (IκB), thus releasing the NF-κB dimer that translocates to the nucleus to exert transcriptional control. The RING domain of E3 ubiquitin ligase TRAF2 binds and activates SphK1, responsible for increased S1P levels essential for NF-κB-dependent p21/cip1 and c-fos gene expression that counteract caspase-8 activation and apoptosis induction. This peculiar molecular mechanism that accounts for S1P pro-survival effects relies exclusively on intracellular effects, since agonists of S1PR (such as dihydro S1P) unable to interact with TRAF2 do not possess anti-apoptotic effects. Moreover, S1P is able to specifically bind to prohibitin 2 (PHB2), regulating mitochondrial respiratory chain assembly. PHB2 is a highly conserved, ubiquitously expressed protein that forms large complexes in the inner mitochondrial membrane composed of heterodimers of PHB2/PHB1. SphK2 located in the mitochondria produces S1P that interacts with PHB2 and affects the assembly of complex IV (COX) respiratory chain activity and oxygen consumption (Strub et al., 2010). Finally, it was reported that both pharmacological inhibition/knockdown of SphK2, and overexpression of S1P lyase/SPP1 in neuroblastoma cells abrogate BACE1-mediated Aβ production (Takasugi et al., 2011) whereas exogenous S1P failed to increase amyloid-beta (Aβ) production. On the other hand, SphK2 overexpression increased the amount of secreted Aβ by producing S1P that interacts with BACE1, suggesting that SphK2-generated S1P regulates Aβ production via BACE1 activation.

Nevertheless, it has been extensively demonstrated that S1PRs have a major role in S1P-dependent biological effects. Importantly depending on the expressed profile of S1PRs, S1P appears capable of differently affecting key cellular events such as proliferation, survival, motility and differentiation in different cell types (Chun et al., 2000; Spiegel and Milstien, 2003; Blaho and Hla, 2011; Soliven et al., 2011). Indeed, S1PR have a wide variety of biological effects in multiple organs and tissues, such as immune, cardiovascular and respiratory systems, as well as in the CNS. Experimental evidence has further highlighted S1P receptors as a potential targets for the regulation of vascular permeability and neuroprotection in different conditions such as pain, stroke and demyelinating diseases (Fyrst and Saba, 2010).

### Multiple Sclerosis and Fingolimod

Myriocin-derivative FTY720 (fingolimod), the active substance of Gilenya^®^, has been authorized by Food and Drug Administration (FDA) and European Medicines Agency (EMA) as the first oral treatment for relapsing-remitting MS, based on extensive clinical trials (Kappos et al., 2010; Cohen et al., 2010). FTY720 is a sphingosine analog prodrug phosphorylated by SphK2 isozyme to produce the active form FTY720-phosphate (FTY720-P) (Paugh et al., 2003; Billich et al., 2003; Zemann et al., 2006; Figure 2), that in turn activates four out of five S1PR receptor subtypes, except S1P_2_, in the range of sub-nanomolar concentrations (Brinkmann and Lynch, 2002; Mandala et al., 2002). As initially reported, FTY720 was found to act as immunomodulator capable of depleting mature T cells in allograft models (Chiba et al., 2005). Although early studies addressed FTY720 as a low-efficacy suppressor of transplantation rejection (Budde et al., 2006), its successful employment in EAE supported the therapeutic action for MS treatment (Brinkmann and Lynch, 2002; Fujino et al., 2003; Webb et al., 2004; Kataoka et al., 2005; Papadopoulos et al., 2010).

The main recognized mechanism by which FTY720 improves MS disease is by affecting immune responses, specifically regulating lymphocyte trafficking. Circulating T lymphocytes express S1P_1_ and lower levels of S1P_3__/__4_ (Graeler and Goetzl, 2002) receptors, and the interaction of exogenous S1P with S1P_1_ is capable of initiating lymphocyte egress from lymph nodes by overcoming retention signals (Brinkmann et al., 2010). Although acute administration of FTY720-P activates S1P_1_ (Camm et al., 2014), chronic exposure to the S1P analogue leads to irreversible receptor internalization resulting in ‘functional antagonism’ of S1P_1_ signaling (Graler and Goetzl, 2004; Brinkmann, 2009; Gergely et al., 2012). FTY720 has a selective mechanism of action, targeting specific subclasses of lymphocytes: FTY720 treatment negatively modulates S1P_1_, thus causing a retention of circulating pathogenic lymphocytes (naive and central memory T cells positive for the chemokine receptor 7, the CCR7) back into the lymph nodes (Matloubian et al., 2004), thereby preventing their infiltration into the CNS, where they exert pathological effects (Cohen and Chun, 2011). However, FTY720 does not significantly affect activation or proliferation of redistributed naïve and central memory T cells and does not block the egress of CCR7-negative effector memory T cells from lymph nodes, preserving immunosurveillance (Mehling et al., 2008).

Intriguingly, differently from FTY720, apoM-S1P is unessential for lymphocyte trafficking although limits lymphopoiesis by activating the S1P_1_ receptor on lymphocyte progenitors. Indeed, the effect exerted by apoM-bound S1P has been investigated in EAE mice lacking apoM, who developed more severe disease with increased lymphocytes in the CNS and breakdown of the BBB. Moreover, apoM-bound S1P, but not albumin-, inhibits lymphopoiesis *in vitro* and overexpression of apoM in rodents decreases endothelial inflammation and EAE manifestation exerting a protective function against autoimmune inflammation (Blaho et al., 2015; Wang et al., 2019).

In addition to immunological actions, FTY720 can penetrate the BBB acting on different S1P receptors that are expressed in brain resident cells, such as astrocytes and oligodendrocytes, claiming the possibility that FTY720 may elicit direct actions on CNS. Indeed, after the treatment with the pro-drug FTY720, FTY720-P has been detected in the cerebrospinal fluid at sub-nanomolar levels (Foster et al., 2007), which are sufficient to modulate human CNS cell properties *in vitro* (Miron et al., 2008c). This approach is consistent with multiple actions of the lysosphingolipid in the CNS, in line with growing literature describing direct effects of S1P signaling on brain cells. Using S1P receptor specific agonists and antagonists, S1P_3_ and S1P_5_ were involved in FTY720-induced effects for the remyelination and astrogliosis, whereas S1P_1_ and S1P_5_ affected microgliosis (Miron et al., 2010). Therapeutic interventions that affect oligodendrocyte remyelination processes could be critical factors for long-term functional recovery in MS. In particular, in organotypic cerebellar slices (Miron et al., 2010), the S1P analog was found to enhance remyelination which occurs subsequently to a demyelinating insult, this event being mediated at least in part by OPC differentiating into myelinating OLs.

Although FTY720 treatment has no positive impact on myelin content under basal conditions, FTY720 administration increases both remyelination score subsequent to lysolecithin-induced damage, and the number of endogenous OPCs within the lesion by facilitating migration, recruitment and proliferation of these cells. Furthermore, it enhances OPC process extension and differentiation into mature OLs (Yazdi et al., 2015), as well as amplifies the number of axons with remyelinated myelin sheaths. Despite these findings, in cuprizone model of MS, FTY720 treatment do not enhance remyelination while decreasing severity of demyelination and promoting OPC proliferation (Kim et al., 2011; Alme et al., 2015). However, in this MS model, fingolimod induces higher number of mature OLs near demyelinated areas, indicating a potential effect on differentiation and/or migration without apparent effects on remyelination (Hu et al., 2011; Alme et al., 2015; Nystad et al., 2020). Nevertheless, another research group reported that fingolimod treatment (0.3 mg/kg) causes remyelination in acute cuprizone-induced demyelination model (Slowik et al., 2015). Hypothesis on such inconsistencies can be made regarding the concentration of fingolimod as well as time-dependence that may cause different effects in cuprizone model. Fingolimod (1 mg/kg) treatment after 3 days from cuprizone administration induces survival of mature OLs whereas later administration of the drug (started at 10 days after cuprizone treatment) lacks cytoprotective effect (Kim et al., 2018), claiming the possibility that early intervention appears to be required to prevent demyelination. In cuprizone model of demyelination, FTY720 (0.3 mg/kg), co-administered with transplanted neural progenitor cells derived from induced pluripotent cells (iPS-NPC), increases OPC survival and proliferation. Moreover, the authors assess that differentiation of transplanted iPS-NPC into oligodendroglial lineage may occur (Yazdi et al., 2015).

Treatment with FTY720 at the onset of EAE reduces clinical symptoms and decreases demyelination by blocking the Akt/mTOR signaling pathway (Hou et al., 2016). In later stage of EAE disease, FTY720 treatment increases MBP level and promotes the appearance of newly generated myelinating OLs, via Sonic Hedgehog signaling pathway, decreases EAE clinical manifestation, and improves neurological functions (Zhang et al., 2015). Furthermore, FTY720 administration (1 mg/kg) during EAE induction in female mice reduces demyelinated area, axonal damage, brain atrophy while increasing brain-derived neurotrophic factor (BDNF) level and clinical scores (Fukumoto et al., 2014; Smith et al., 2018), underling the positive effect of FTY720 on regeneration in different types of demyelination models.

Finally, conditional knockout of S1P_1_ in neural lineages have been used to identify a key role for astrocytes in reducing the severity of pathological changes in EAE. Indeed, the efficacy of fingolimod is lost by astrocytic deletion of S1P_1_, highlighting that the main protective effect of this compound in EAE comprises the modulation of astrocyte function by S1P_1_ (Choi et al., 2011).

### Sphingosine 1-Phosphate Receptor Signaling in Oligodendrocytes and Interaction With A_2__*B*_Rs

OPC availability, recruitment and differentiation into mature oligodendrocytes are pivotal aspects involved in remyelination. Experimental evidence demonstrates that S1PRs display different biological effects depending on oligodendrocyte differentiation state. Oligodendroglial cells express four out of five S1PRs, S1P_1_, S1P_2_, S1P_3_, and S1P_5_, whose expression profiles change and are selectively controlled during oligodendrogenesis. Indeed, the predominant isoform in immature OPC is S1P_1_ whereas S1P_2_, S1P_3_ and S1P_5_ are present at much lower levels (Im et al., 2000; Yu N. C. et al., 2004; Jaillard et al., 2005; Novgorodov et al., 2007). During differentiation from OPC into mature OLs, S1P_5_ and S1P_1_ are reciprocally regulated, the first being augmented while the latter decreases (Jung et al., 2007; Coelho et al., 2007; Miron et al., 2008c; van Doom et al., 2012). Indeed, human mature OLs express S1PR transcripts in relative abundance of S1P_5_ > S1P_3_ > S1P_1_ (Miron et al., 2008a). In particular, the modulation of S1PR expression seems to be mediated by PDGF that is produced by OPCs. Indeed, S1PRs are differentially modulated by PDGF resulting in downregulation of S1P_5_ and upregulation of S1P_1_ in OPCs. Downregulation of S1P_1_ by RNA interference affects PDGF-induced proliferation of OPCs (Jung et al., 2007) whereas, in mature OLs, S1P signaling promotes cell survival, suggesting that S1PRs may exert different functions during oligodendroglial development (Okada et al., 2009). The effects of fingolimod on oligodendrocyte process dynamics depended on both dose and duration of treatment, since S1PR activation by fingolimod induces process retraction in O4-positive pre-OLs, that appears to be transient and restricted to immature cells, not observed at later and mature developmental stages (Miron et al., 2008b). Process retraction is mediated by S1P_5_ *via* a ROCK/collapsin response-mediated signaling pathway, whereas the effect is abrogated in S1P_5_^–/–^ mice derived oligodendrocytes. Prolonged treatment with higher doses of fingolimod induces process extension associated with Rac1-linked cytoskeletal signaling cascades mimicked by a S1P_1_ agonist SEW2871 (Miron et al., 2008b). Similarly, these results can be corroborated by the fact that, depending on the dose, FTY720-P exerts opposite actions in rat OPC cultures (Jung et al., 2007). Indeed, high concentration of FTY720-P (1 μM) inhibits, OPC differentiation, whereas low concentration (10 nM) enhances both the percentage of mature OLs and MBP expression.

Furthermore, S1P-induced survival of mature OLs is mediated through a pertussis toxin- (PTx) sensitive, Akt-mediated pathway, since the molecular mechanism activated by S1PRs in oligodendrocytes specifically involves the phosphorylation of ERK1/2 and Akt, that subsequently promotes cell survival (Jaillard et al., 2005; van Doom et al., 2012). Noteworthy, the pro-survival effect of S1PR in human mature OLs is mimicked by the administration of S1P_5_ agonist. Interestingly, S1PR agonists affect oligodendrocytes differentiation stages depending on the molecular mechanisms evoked and in a concentration-dependent manner. In particular, human OPC cultures treated with low nanomolar concentrations of either FTY720-P or S1P present enhanced differentiation into both pre-OLs (O4-positive) and mature (GC-positive) OLs. Of note, ERK1/2 pathway is involved in the differentiation of OPC into O4 positive-cells, since inhibition of MAPK pathway by U0126 prevented pre-OLs generation. However, the transition to mature OLs is mediated by p38MAPK signaling, since PD169316 administration blocks the progression of O4 positive into mature stages of differentiation (Cui et al., 2014).

Notably, experimental evidences highlight that activation of S1P_5_ induces process retraction whereas activation of S1P_1_ enhances process extension of OPCs (Miron et al., 2008b), and coherently during early myelination S1P_1_ abrogation in the oligodendroglial cells delays OPC differentiation into mature OLs (Kim et al., 2011). In agreement, S1P1^–/–^ mice show decreased myelin protein expression, thinner myelin and susceptibility to demyelination induced by cuprizone, that is conceivable with the major expression of S1P_1_ in OPCs that could play a pivotal role in early OPC differentiation stages. On the contrary, abnormalities in myelination are not evident in S1P_5_^–/–^ mice; oligodendrocytes display a higher expression of S1P_5_ compared to other S1PRs where it may be involved in late myelination processes. Although S1P_5_ is expressed at lower levels in OPCs compared to OLs and OPCs are devoid of pro-survival effects of S1P_5_, this receptor subtype is activated by S1P to arrest OPC migration (Novgorodov et al., 2007). Indeed, S1P-induced decrease of chemiotaxis is completely prevented by the specific downregulation of S1P_5_, but not S1P_2_, and is insensitive to PTx, suggesting that S1P_5_-initiated signaling is not mediated by the Gα_*i*_-protein coupled pathway. The molecular mechanism that is responsible for the impairment of migratory capacity after S1P administration involves G_12__/__13_ that stimulates the Rho/ROCK signaling pathway (Novgorodov et al., 2007). The authors suggest that glutamate treatment of OPC may increase S1P export and S1P extracellular levels to modulate OPC motility and claim the possibility for S1P to be a part of the neuron-oligodendroglial communication network in developing brain, that could have a role also during remyelination processes. These data are also consistent with the physiological effect of S1P_5_ in OPCs during differentiation postulating that changes in receptor coupling with heterotrimeric G-proteins may occur, thus leading to the activation of different signaling pathways.

Finally, SphK1 has a protective role against apoptosis in OPC cultures (Saini et al., 2005). In particular, Neurotrophin 3 (NT-3) accounts for SphK1 translocation to plasma membrane and activation to exert its pro-survival effect in these cells. Remarkably, down-regulation of SphK1 negatively affects the capacity of NT-3 to protect oligodendrocyte progenitors from apoptosis. In agreement with these findings, analysis of plaques from MS brains shows reduced levels of S1P and increased sphingosine and C16/18-ceramide (Qin et al., 2010).

**FIGURE 4:**
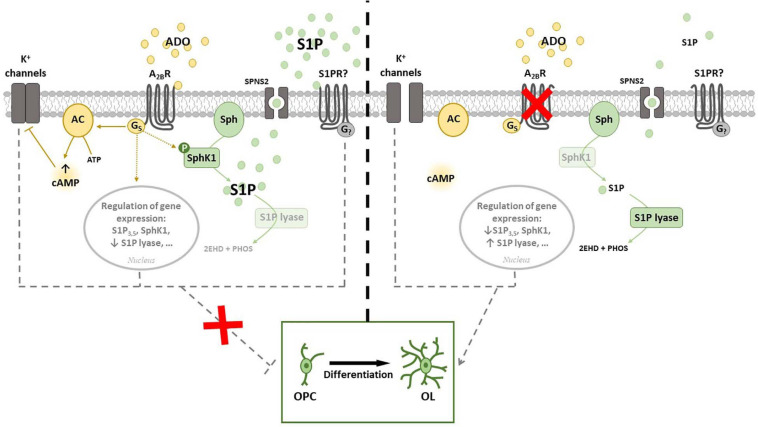
Cross-talk between adenosine A_2__*B*_ receptor (A_2__*B*_R) and Sphingosine 1-phosphate (S1P). (**Left panel)** A_2__*B*_R throughout G_*s*_ activation leads to an increase of intracellular cAMP that inhibits K^+^ channels. The activation of A_2__*B*_R regulates gene expression of proteins involved in S1P signaling and metabolism and increases the phosphorylation of SphK1. All these events prevent OPC differentiation *in vitro***. (Right panel)** A_2__*B*_R silencing allows K^+^ channels to be opened and strikingly increases expression of S1P lyase, that irreversible catalyzes S1P and blocks S1P degradation signaling. In this conditions OPC differentiation is facilitated. Abbreviations: Adenosine (ADO); Sphingosine (Sph) kinase 1 (SphK1); specific spinster homolog 2 (SPNS2); adenylyl cyclase (AC) (Coppi et al., 2020a,b).

In the light of above results, we recently performed a study aimed to merge current knowledge on S1P pathway with A_2__*B*_R signaling. Namely, by using either or both pharmacological approach and receptor silencing, we demonstrated that an interplay occurs between A_2__*B*_Rs and SphK/S1P axis in OPCs (Figure 4). Indeed, SphK1 enzyme is activated when OPCs are cultured in the presence of the A_2__*B*_R selective agonist BAY60-6583, thus rising S1P production, whereas its silencing by small interference RNA (siRNA) increases the expression of S1P lyase, promoting S1P removal (Coppi et al., 2020a). This observation led to hypothesize that the anti-differentiating effect exerted by A_2__*B*_R activation in OPCs is mediated by an increase in S1P intracellular levels (Figure 4). This hypothesis was confirmed by findings that the SphK inhibitors VPC96047 or VPC96091 markedly increased MAG and MBP expression in OPC cultures, indicating enhanced cell maturation, and also significantly increased I_*K*_ currents, necessary to OPC differentiation (Coppi et al., 2020a). Thus, it appears that an increase in S1P production possibly accounts for the anti-differentiating effect of BAY60-6583 in OPCs, whereas A_2__*B*_R silencing, by promoting S1P removal through the activation of S1P lyase, facilitates OPC maturation. An additional proof that A_2__*B*_Rs and S1P signaling are interconnected in OPCs resides in the fact that this receptor subtype is upregulated during cell differentiation, an effect that is completely prevented when cells are differentiated in the presence of the pan SphK inhibitor VPC96047 (Coppi et al., 2020a).

We also found that FTY720-P differently affects BAY60-6583-mediated K^+^ current inhibition depending upon the concentration applied. When administered at 1 μM, it mimicked and partially occluded the effect of a subsequent BAY60-6583 application on voltage-dependent K^+^ currents. This confirms, again, that S1P and A_2__*B*_R pathways converge. On the other hand, the effect of BAY60-6583 on ramp currents was significantly enhanced in the presence of low (10 nM) FTY720-P (Coppi et al., 2020a). Similarly, 10 nM FTY720-P increased, whereas 1 μM decreased, MAG expression after 7 days of OPC maturation. Contrasting effects of this compound depending on the concentration used have been previously reported by others, the effects of fingolimod depending on both concentration and treatment duration (Jung et al., 2007; Miron et al., 2008c).

Differently from FTY720-P, when S1P is used as a ligand, the effect on K^+^ currents was not observed (Coppi et al., 2020a). This apparent discrepancy may be ascribed to the fact that receptor ligation by FTY720-P is restricted to all S1P receptors except S1P_2_, which however is activated by S1P. Moreover, the functional outcome induced by S1PR modulators could differ from one ligand to another since it could differently affect receptor fate. Indeed, it has been shown that FTY720-P can induce S1P_1_ receptor degradation, whereas S1P affects receptor recycling. Finally the lack of effect exerted by exogenous S1P on I_*K*_ is in agreement with previous data in different cellular models, such as skeletal muscle cells, where agonist-induced S1P intracellular production and activity, called inside-out signaling, has different, even sometimes opposite, actions compared to exogenous S1P (Donati et al., 2004; Cencetti et al., 2010; Cencetti et al., 2013). This effect can be explained by a localized release of bioactive lipid in membrane microdomain where the availability of certain receptor subtypes is limited. The spatial regulation of S1P biosynthesis within the cell, together with its localized partitioning into plasma membrane domains, determines the subset of engaged S1PRs and thus the biological outcome (Donati et al., 2013).

## Conclusion

Multiple evidences indicate that adenosine may interact with S1P signaling in orchestrating the processes concurring to OPC maturation and thus to the remodeling of brain development and/or repair after a demyelinating insult.

In particular, adenosine A_2__*B*_R appears to play a critical role in oligodendrogliogenesis since its agonism activates SphK1 and reduces OPC differentiation. These data are in agreement with the fact that, on one hand, SphK inhibition decreases A_2__*B*_R expression leading to an increase in OPC differentiation, and on the other, specific down-regulation of A_2__*B*_R reduces SphK1 and potently induces S1P lyase expression thus pushing S1P toward catabolism, and thereby facilitating OPC maturation. On the whole, the available results suggest that A_2__*B*_R antagonism represents a possible co-adjuvant strategy to improve remyelination promoted by the prodrug sphingosine analog, FTY720 (Fingolimod) for the treatment of MS, as confirmed by previous findings in the literature reporting a protective role of A_2B_R block in EAE models (Wei et al., 2013; Liu et al., 2020).

## Author Contributions

EC, FP, PB, AP, and FCe: conceptualization. FP and PB validation. EC, AP, PB, FP, FCe, and CD: resources. EC, FCe, FCh, and AP: writing–original draft preparation. MV, FCh, FCe, and CD writing–review and editing. All authors contributed to the article and approved the submitted version.

## Conflict of Interest

The authors declare that the research was conducted in the absence of any commercial or financial relationships that could be construed as a potential conflict of interest.

## References

[B1] Alemany R. van Koppen C. J. Danneberg K. ter Braak M. Heringdorf D. M. Z. (2007). Regulation and functional roles of sphingosine kinases. *Naunyn Schmiedeb. Arch. Pharmacol.* 374 413–428. 10.1007/s00210-007-0132-3 17242884

[B2] Allende M. L. Sasaki T. Kawai H. Olivera A. Mi Y. van Echten-Deckert G. (2004). Mice deficient in sphingosine kinase 1 are rendered lymphopenic by FTY720. *J. Biol. Chem.* 279 52487–52492. 10.1074/jbc.m406512200 15459201

[B3] Alme M. N. Nystad A. E. Bo L. Myhr K. M. Vedeler C. A. Wergeland S. (2015). Fingolimod does not enhance cerebellar remyelination in the cuprizone model. *J. Neuroimmunol.* 285 180–186. 10.1016/j.jneuroim.2015.06.006 26198937

[B4] Alvarez S. E. Harikumar K. B. Hait N. C. Allegood J. Strub M. G. Kim Y. E. (2010). Sphingosine-1-phosphate is a missing cofactor for the E3 ubiquitin ligase TRAF2. *Nature* 465 1084–U1149. 20577214 10.1038/nature09128 PMC2946785

[B5] Antonioli L. Blandizzi C. Pacher P. Hasko G. (2013). Immunity, inflammation and cancer: a leading role for adenosine. *Nat. Rev. Cancer* 13 842–857. 10.1038/nrc3613 24226193

[B6] Antonioli L. Blandizzi C. Pacher P. Hasko G. (2019). The purinergic system as a pharmacological target for the treatment of immune-mediated inflammatory diseases. *Pharmacol. Rev.* 71 345–382. 10.1124/pr.117.014878 31235653 PMC6592405

[B7] Asghari A. A. Azarnia M. Mirnajafi-Zadeh J. Javan M. (2013). Adenosine A1 receptor agonist, N6-cyclohexyladenosine, protects myelin and induces remyelination in an experimental model of rat optic chiasm demyelination; electrophysiological and histopathological studies. *J. Neurol. Sci.* 325 22–28. 10.1016/j.jns.2012.11.008 23260322

[B8] Attali B. Wang N. Kolot A. Sobko A. Cherepanov V. Soliven B. (1997). Characterization of delayed rectifier Kv channels in oligodendrocytes and progenitor cells. *J. Neurosci.* 17 8234–8245. 10.1523/jneurosci.17-21-08234.1997 9334399 PMC6573763

[B9] Barateiro A. Fernandes A. (2014). Temporal oligodendrocyte lineage progression: in vitro models of proliferation, differentiation and myelination. *Biochim. Biophys. Acta* 1843 1917–1929. 10.1016/j.bbamcr.2014.04.018 24768715

[B10] Barres B. A. Koroshetz W. J. Swartz K. J. Chun L. L. Y. Corey D. P. (1990). Ion channel expression by white matter glia - the o-2a glial progenitor-cell. *Neuron* 4 507–524. 10.1016/0896-6273(90)90109-s 1691005

[B11] Bergles D. E. Richardson W. D. (2016). Oligodendrocyte development and plasticity. *Cold Spring Harb. Perspect. Biol.* 8:a020453. 10.1101/cshperspect.a020453 26492571 PMC4743079

[B12] Bernacchioni C. Cencetti F. Blescia S. Donati C. Bruni P. (2012). Sphingosine kinase/sphingosine 1-phosphate axis: a new player for insulin-like growth factor-1-induced myoblast differentiation. *Skelet. Muscle* 2:15. 10.1186/2044-5040-2-15 22788716 PMC3439699

[B13] Bernacchioni C. Ghini V. Squecco R. Idrizaj E. Garella R. Puliti E. (2021). Role of sphingosine 1-phosphate signalling axis in muscle atrophy induced by TNF alpha in C2C12 myotubes. *Int. J. Mol. Sci.* 22:1280. 10.3390/ijms22031280 33525436 PMC7866171

[B14] Betti M. Catarzi D. Varano F. Falsini M. Varani K. Vincenzi F. (2018). The aminopyridine-3,5-dicarbonitrile core for the design of new non-nucleoside-like agonists of the human adenosine A(2B) receptor. *Eur. J. Med. Chem.* 150 127–139. 10.1016/j.ejmech.2018.02.081 29525433

[B15] Billich A. Bornancin F. Devay P. Mechtcheriakova D. Urtz N. Baumruker T. (2003). Phosphorylation of the immunomodulatory drug FTY720 by sphingosine kinases. *J. Biol. Chem.* 278 47408–47415. 10.1074/jbc.m307687200 13129923

[B16] Blaho V. A. Galvani S. Engelbrecht E. Liu C. Swendeman S. L. Kono M. (2015). HDL-bound sphingosine-1-phosphate restrains lymphopoiesis and neuroinflammation. *Nature* 523:342. 10.1038/nature14462 26053123 PMC4506268

[B17] Blaho V. A. Hla T. (2011). Regulation of mammalian physiology, development, and disease by the Sphingosine 1-Phosphate and Lysophosphatidic acid receptors. *Chem. Rev.* 111 6299–6320. 10.1021/cr200273u 21939239 PMC3216694

[B18] Bonetto G. Kamen Y. Evans K. A. Karadottir R. T. (2020). Unraveling myelin plasticity. *Front. Cell. Neurosci.* 14:156. 10.3389/fncel.2020.00156 32595455 PMC7301701

[B19] Bortoluzzi A. Vincenzi F. Govoni M. Padovan M. Ravani A. Borea P. A. (2016). A(2A) adenosine receptor upregulation correlates with disease activity in patients with systemic lupus erythematosus. *Arthrit. Res. Ther.* 18:192. 10.1186/s13075-016-1089-8 PMC5002091 27566294

[B20] Brazel C. Y. Rosti R. T. Boyce S. Rothstein R. P. Levison S. W. (2004). Perinatal hypoxia/ischemia damages and depletes progenitors from the mouse subventricular zone. *Dev. Neurosci.* 26 266–274. 10.1159/000082143 15711066 PMC1343454

[B21] Brinkmann V. (2009). FTY720 (fingolimod) in multiple sclerosis: therapeutic effects in the immune and the central nervous system. *Br. J. Pharmacol.* 158 1173–1182. 10.1111/j.1476-5381.2009.00451.x 19814729 PMC2782328

[B22] Brinkmann V. Billich A. Baumruker T. Heining P. Schmouder R. Francis G. (2010). Fingolimod (FTY720): discovery and development of an oral drug to treat multiple sclerosis. *Nat. Rev. Drug Discov.* 9 883–897. 10.1038/nrd3248 21031003

[B23] Brinkmann V. Lynch K. R. (2002). FTY720: targeting G-protein-coupled receptors for sphingosine 1-phosphate in transplantation and autoimmunity. *Curr. Opin. Immunol.* 14 569–575. 10.1016/s0952-7915(02)00374-6 12183155

[B24] Bruno G. Cencetti F. Pini A. Tondo A. Cuzzubbo D. Fontant F. (2020). beta 3-adrenoreceptor blockade reduces tumor growth and increases neuronal differentiation in neuroblastoma via SK2/S1P(2) modulation. *Oncogene* 39 368–384. 10.1038/s41388-019-0993-1 31477835 PMC6949192

[B25] Budde K. Schutz M. Glander P. Peters H. Waiser J. Liefeldt L. (2006). FTY720 (fingolimod) in renal transplantation. *Clin. Transplant.* 20 17–24. 10.1111/j.1399-0012.2006.00596.x 17100697

[B26] Burnstock G. Fredholm B. B. Verkhratsky A. (2011). Adenosine and ATP receptors in the brain. *Curr. Top. Med. Chem.* 11 973–1011. 10.2174/156802611795347627 21401499

[B27] Camm J. Hla T. Bakshi R. Brinkmann V. (2014). Cardiac and vascular effects of fingolimod: mechanistic basis and clinical implications. *Am. Heart J.* 168 632–644. 10.1016/j.ahj.2014.06.028 25440790

[B28] Cartier A. Hla T. (2019). Sphingosine 1-phosphate: lipid signaling in pathology and therapy. *Science* 366:323. 10.1126/science.aar5551 PMC7661103 31624181

[B29] Cencetti F. Bernacchioni C. Nincheri P. Donati C. Bruni P. (2010). Transforming growth factor-beta 1 induces transdifferentiation of myoblasts into myofibroblasts via up-regulation of sphingosine Kinase-1/S1P(3) axis. *Mol. Biol. Cell* 21 1111–1124. 10.1091/mbc.e09-09-0812 20089836 PMC2836962

[B30] Cencetti F. Bernacchioni C. Tonelli F. Roberts E. Donati C. Bruni P. (2013). TGF beta 1 evokes myoblast apoptotic response via a novel signaling pathway involving S1P(4) transactivation upstream of Rho-kinase-2 activation. *FASEB J.* 27 4532–4546. 10.1096/fj.13-228528 23913862

[B31] Chandrasekaran B. Samarneh S. Jaber A. M. Y. Kassab G. Agrawal N. (2019). Therapeutic potentials of A(2B) adenosine receptor ligands: current status and perspectives. *Curr. Pharm. Des.* 25 2741–2771. 10.2174/1381612825666190717105834 31333084

[B32] Chang A. Tourtellotte W. W. Rudick R. Trapp B. D. (2002). Premyelinating oligodendrocytes in chronic lesions of multiple sclerosis. *N. Engl. J. Med.* 346 165–173. 10.1056/nejmoa010994 11796850

[B33] Chen G. Q. Chen Y. Y. Wang X. S. Wu Z. S. Yang M. H. Xu H. Q. (2010). Chronic caffeine treatment attenuates experimental autoimmune encephalomyelitis induced by guinea pig spinal cord homogenates in Wistar rats. *Brain Res.* 1309 116–125. 10.1016/j.brainres.2009.10.054 19879252

[B34] Chen J. F. Eltzschig H. K. Fredholm B. B. (2013). Adenosine receptors as drug targets - what are the challenges? *Nat. Rev. Drug Discov.* 12 265–286. 10.1038/nrd3955 23535933 PMC3930074

[B35] Cherchi F. Pugliese A. M. Coppi E. (2021). Oligodendrocyte precursor cell maturation: role of adenosine receptors. *Neural Regen. Res.* 16 1686–1692. 10.4103/1673-5374.306058 33510056 PMC8328763

[B36] Chiba K. Hoshino Y. Ohtsuki M. Kataoka H. Maeda Y. Matsuyuki H. (2005). Immunosuppressive activity of FTY720, sphingosine 1-phosphate receptor agonist: I. prevention of allograft rejection in rats and dogs by FTY720 and FTY720-phosphate. *Transplant. Proc.* 37 102–106. 10.1016/j.transproceed.2004.12.286 15808561

[B37] Chittajallu R. Aguirre A. A. Gallo V. (2005). Downregulation of platelet-derived growth factor-alpha receptor-mediated tyrosine kinase activity as a cellular mechanism for K+ channel regulation during oligodendrocyte development in situ. *J. Neurosci.* 25 8601–8610. 10.1523/jneurosci.2122-05.2005 16177027 PMC6725520

[B38] Choi J. W. Gardell S. E. Herr D. R. Rivera R. Lee C. Noguchi K. (2011). FTY720 (fingolimod) efficacy in an animal model of multiple sclerosis requires astrocyte sphingosine 1-phosphate receptor 1 (S1P(1)) modulation. *Proc. Natl. Acad. Sci. U.S.A.* 108 751–756. 10.1073/pnas.1014154108 21177428 PMC3021041

[B39] Christensen P. M. Liu C. H. Swendeman S. L. Obinata H. Qvortrup K. Nielsen L. B. (2016). Impaired endothelial barrier function in apolipoprotein M-deficient mice is dependent on sphingosine-1-phosphate receptor 1. *FASEB J.* 30 2351–2359. 10.1096/fj.201500064 26956418 PMC4871798

[B40] Christoffersen C. Obinata H. Kumaraswamy S. B. Galvani S. Ahnström J. Sevvana M. (2011). Endothelium-protective sphingosine-1-phosphate provided by HDL-associated apolipoprotein M. *Proc. Natl. Acad. Sci. U.S.A.* 108 9613–9618. 10.1073/pnas.1103187108 21606363 PMC3111292

[B41] Chun J. Weiner J. A. Fukushima N. Contos J. J. Zhang G. Kimura Y. (2000). Neurobiology of receptor-mediated lysophospholipid signaling - From the first lysophospholipid receptor to roles in nervous system function and development. *Ann. N. Y. Acad. Sci.* 905 110–117. 10.1111/j.1749-6632.2000.tb06543.x 10818447

[B42] Ciana P. Fumagalli M. Trincavelli M. L. Verderio C. Rosa P. Lecca D. (2006). The orphan receptor GPR17 identified as a new dual uracil nucleotides/cysteinyl-leukotrienes receptor. *EMBO J.* 25 4615–4627. 10.1038/sj.emboj.7601341 16990797 PMC1589991

[B43] Coelho R. P. Payne S. G. Bittman R. Spiegel S. Sato-Bigbee C. (2007). The immunomodulator FTY720 has a direct cytoprotective effect in oligodendrocyte progenitors. *J. Pharmacol. Exp. Ther.* 323 626–635. 10.1124/jpet.107.123927 17726159

[B44] Cohen J. A. Barkhof F. Comi G. Hartung H. Khatri O. B. Montalban X. (2010). Oral fingolimod or intramuscular interferon for relapsing multiple sclerosis. *N. Engl. J. Med.* 362 402–415. 20089954 10.1056/NEJMoa0907839

[B45] Cohen J. A. Chun J. (2011). Mechanisms of Fingolimod’s efficacy and adverse effects in multiple sclerosis. *Ann. Neurol.* 69 759–777. 10.1002/ana.22426 21520239

[B46] Colotta V. Lenzi O. Catarzi D. Varano F. Squarcialupi L. Costagli C. (2012). 3-Hydroxy-1H-quinazoline-2,4-dione derivatives as new antagonists at ionotropic glutamate receptors: molecular modeling and pharmacological studies. *Eur. J. Med. Chem.* 54 470–482. 10.1016/j.ejmech.2012.05.036 22704999

[B47] Constantinescu C. S. Farooqi N. O’Brien K. Gran B. (2011). Experimental autoimmune encephalomyelitis (EAE) as a model for multiple sclerosis (MS). *Br. J. Pharmacol.* 164 1079–1106. 10.1111/j.1476-5381.2011.01302.x 21371012 PMC3229753

[B48] Coppi E. Cellai L. Maraula G. Dettori I. Melani A. Pugliese A. M. (2015). Role of adenosine in oligodendrocyte precursor maturation. *Front. Cell. Neurosci.* 9:155. 10.3389/fncel.2015.00155 25964740 PMC4408841

[B49] Coppi E. Cellai L. Maraula G. Pugliese A. M. Pedata F. (2013a). Adenosine A(2A) receptors inhibit delayed rectifier potassium currents and cell differentiation in primary purified oligodendrocyte cultures. *Neuropharmacology* 73 301–310. 10.1016/j.neuropharm.2013.05.035 23770463

[B50] Coppi E. Maraula G. Fumagalli M. Failli P. Cellai L. Bonfanti E. (2013b). UDP-glucose enhances outward K plus currents necessary for cell differentiation and stimulates cell migration by activating the GPR17 receptor in oligodendrocyte precursors. *Glia* 61 1155–1171. 10.1002/glia.22506 23640798

[B51] Coppi E. Cherchi F. Fusco I. Dettori I. Gaviano L. Magni G. (2020a). Adenosine A(2B) receptors inhibit K+ currents and cell differentiation in cultured oligodendrocyte precursor cells and modulate sphingosine-1-phosphate signaling pathway. *Biochem. Pharmacol.* 177:113956. 10.1016/j.bcp.2020.113956 32251679

[B52] Coppi E. Dettori I. Cherchi F. Bulli I. Venturini M. Lana D. (2020b). A(2B) adenosine receptors: when outsiders may become an attractive target to treat brain ischemia or Demyelination. *Int. J. Mol. Sci.* 21:9697. 10.3390/ijms21249697 33353217 PMC7766015

[B53] Coppi E. Dettori I. Cherchi F. Bulli I. Venturini M. Pedata F. (2021). New insight into the role of adenosine in demyelination, stroke and neuropathic pain. *Front. Pharmacol.* 11:625662. 10.3389/fphar.2020.625662 33584309 PMC7878385

[B54] Coppi E. Pedata F. Gibb A. J. (2012). P2Y(1) receptor modulation of Ca2+-activated K+ currents in medium-sized neurons from neonatal rat striatal slices. *J. Neurophysiol.* 107 1009–1021. 10.1152/jn.00816.2009 22131374 PMC3289470

[B55] Corradetti R. Lo Conte G. Moroni F. Passani B. M. Pepeu G. (1984). Adenosine decreases aspartate and glutamate release from rat hippocampal slices. *Eur. J. Pharmacol.* 104 19–26. 10.1016/0014-2999(84)90364-9 6149943

[B56] Courtney A. M. Treadaway K. Remington G. Frohman E. (2009). Multiple sclerosis. *Med. Clin. North Am.* 93 451–476. 19272518 10.1016/j.mcna.2008.09.014

[B57] Cui Q. L. Fang J. Kennedy T. E. Almazan G. Antel J. P. (2014). Role of p38MAPK in S1P receptor-mediated differentiation of human oligodendrocyte progenitors. *Glia* 62 1361–1375. 10.1002/glia.22688 24810969

[B58] Cyster J. G. Schwab S. R. (2012). Sphingosine-1-phosphate and lymphocyte egress from lymphoid organs. *Ann. Rev. Immunol.* 30 69–94. 10.1146/annurev-immunol-020711-075011 22149932

[B59] Dawson M. R. L. Polito A. Levine J. M. Reynolds R. (2003). NG2-expressing glial progenitor cells: an abundant and widespread population of cycling cells in the adult rat CNS. *Mol. Cell. Neurosci.* 24 476–488. 10.1016/s1044-7431(03)00210-0 14572468

[B60] de Castro F. Bribian A. (2005). The molecular orchestra of the migration of oligodendrocyte precursors during development. *Brain Res. Rev.* 49 227–241. 10.1016/j.brainresrev.2004.12.034 16111552

[B61] Donati C. Cencetti F. Bruni P. (2013). New insights into the role of sphingosine 1-phosphate and lysophosphatidic acid in the regulation of skeletal muscle cell biology. *Biochim. Biophys. Acta* 1831 176–184. 10.1016/j.bbalip.2012.06.013 22877992

[B62] Donati C. Meacci E. Nuti F. Becciolini L. Farnararo M. Bruni P. (2004). Sphingosine 1-phosphate regulates myogenic differentiation: a major role for S1P(2) receptor. *FASEB J.* 18 449–451. 10.1096/fj.04-1780fje 15625079

[B63] Donati C. Nincheri P. Cencetti F. Rapizzi E. Farnararo M. Bruni P. (2007). Tumor necrosis factor-alpha exerts pro-myogenic action in C2C12 myoblasts dvia sphingosine kinase/S1P(2) signaling. *FEBS Lett.* 581 4384–4388. 10.1016/j.febslet.2007.08.007 17719579

[B64] Dunwiddie T. V. (1984). Interactions between the effects of adenosine and calcium on synaptic responses in rat hippocampus in vitro. *J. Physiol.* 350 545–559. 10.1113/jphysiol.1984.sp015217 6086898 PMC1199285

[B65] Eckle T. Faigle M. Grenz A. Laucher S. Thompson L. F. Eltzschig H. K. (2008). A2B adenosine receptor dampens hypoxia-induced vascular leak. *Blood* 111 2024–2035. 10.1182/blood-2007-10-117044 18056839 PMC2739365

[B66] Emery B. (2010). Regulation of oligodendrocyte differentiation and Myelination. *Science* 330 779–782. 10.1126/science.1190927 21051629

[B67] Feoktistov I. Polosa R. Holgate S. T. Biaggioni I. (1998). Adenosine A(2B) receptors: a novel therapeutic target in asthma? *Trends Pharmacol. Sci.* 19 148–153. 10.1016/s0165-6147(98)01179-1 9612090

[B68] Fields R. D. (2004). Volume transmission in activity-dependent regulation of myelinating glia. *Neurochem. Int.* 45 503–509. 10.1016/j.neuint.2003.11.015 15186916

[B69] Fields R. D. Burnstock G. (2006). Purinergic signalling in neuron-glia interactions. *Nat. Rev. Neurosci.* 7 423–436. 16715052 10.1038/nrn1928 PMC2062484

[B70] Fields R. D. Stevens-Graham B. (2002). Neuroscience - new insights into neuron-glia communication. *Science* 298 556–562. 10.1126/science.298.5593.556 12386325 PMC1226318

[B71] Foster C. A. Howard L. M. Schweitzer A. Persohn E. Hiestand P. C. Balatoni B. (2007). Brain penetraGessiion of the oral immunomodulatory drug FTY720 and its phosphorylation in the central nervous system during experimental autoimmune encephalomyelitis: consequences for mode of action in multiple sclerosis. *J. Pharmacol. Exp. Ther.* 323 469–476. 10.1124/jpet.107.127183 17682127

[B72] Franklin R. J. M. Ffrench-Constant C. (2017). Regenerating CNS myelin - from mechanisms to experimental medicines. *Nat. Rev. Neurosci.* 18 753–769. 10.1038/nrn.2017.136 29142295

[B73] Fredholm B. B. Ijzerman A. P. Jacobson K. A. Linden J. Muller C. E. (2011). International union of basic and clinical pharmacology. LXXXI. nomenclature and classification of adenosine receptors-an update. *Pharmacol. Rev.* 63 1–34. 10.1124/pr.110.003285 21303899 PMC3061413

[B74] Fujino M. Funeshima N. Kitazawa Y. Kimura H. Amemiya H. Suzuki S. (2003). Amelioration of experimental autoimmune encephalomyelitis in lewis rats by FTY720 treatment. *J. Pharmacol. Exp. Ther.* 305 70–77. 10.1124/jpet.102.045658 12649354

[B75] Fukumoto K. Mizoguchi H. Takeuchi H. Horiuchi H. Kawanokuchi J. Jin S. J. (2014). Fingolimod increases brain-derived neurotrophic factor levels and ameliorates amyloid beta-induced memory impairment. *Behav. Brain Res.* 268 88–93. 10.1016/j.bbr.2014.03.046 24713151

[B76] Fumagalli M. Daniele S. Lecca D. Lee P. R. Parravicini C. Fields D. R. (2011). Phenotypic changes, signaling pathway, and functional correlates of GPR17-expressing neural precursor cells during oligodendrocyte differentiation. *J. Biol. Chem.* 286 10593–10604. 10.1074/jbc.m110.162867 21209081 PMC3060511

[B77] Fusco I. Cherchi F. Catarzi D. Colotta V. Varano F. Pedata F. (2019). Functional characterization of a novel adenosine A(2B) receptor agonist on short-term plasticity and synaptic inhibition during oxygen and glucose deprivation in the rat CA1 hippocampus. *Brain Res. Bull.* 151 174–180. 10.1016/j.brainresbull.2019.05.018 31132418

[B78] Fusco I. Ugolini F. Lana D. Coppi E. Dettori I. Gaviano L. (2018). The selective antagonism of adenosine A(2B) receptors reduces the synaptic failure and neuronal death induced by oxygen and glucose deprivation in rat CA1 hippocampus in vitro. *Front. Pharmacol.* 9:399. 10.3389/fphar.2018.00399 29740323 PMC5928446

[B79] Fyrst H. Saba J. D. (2010). An update on sphingosine-1-phosphate and other sphingolipid mediators. *Nat. Chem. Biol.* 6 489–497. 10.1038/nchembio.392 20559316 PMC3001344

[B80] Gallo V. Zhou J. M. McBain C. J. Wright P. Knutson P. L. Armstrong R. C. (1996). Oligodendrocyte progenitor cell proliferation and lineage progression are regulated by glutamate receptor-mediated K+ channel block. *J. Neurosci.* 16 2659–2670. 10.1523/jneurosci.16-08-02659.1996 8786442 PMC6578780

[B81] Galvani S. Sanson M. Blaho V. A. Swendeman S. L. Obinata H. Conger H. (2015). HDL-bound sphingosine 1-phosphate acts as a biased agonist for the endothelial cell receptor S1P(1) to limit vascular inflammation. *Sci. Signal.* 8:ra79. 10.1126/scisignal.aaa2581 26268607 PMC4768813

[B82] Gao P. Peterson Y. K. Smith R. A. Smith C. D. (2012). Characterization of isoenzyme-selective inhibitors of human sphingosine kinases. *PLoS One* 7:e011543. 10.1371/journal.pone.0044543 22970244 PMC3438171

[B83] Gard A. L. Pfeiffer S. E. (1990). 2 proliferative stages of the oligodendrocyte lineage (a2b5+o4- and o4+galc-) under different mitogenic control. *Neuron* 5 615–625. 10.1016/0896-6273(90)90216-3 2223090

[B84] Gault C. R. Obeid L. M. Hannun Y. A. (2010). An overview of sphingolipid metabolism: from synthesis to breakdown. *Adv. Exp. Med. Biol.* 688 1–23. 10.1007/978-1-4419-6741-1_1 20919643 PMC3069696

[B85] Gautier H. O. B. Evans K. A. Volbracht K. James R. Sitnikov S. Lundgaard I. (2015). Neuronal activity regulates remyelination via glutamate signalling to oligodendrocyte progenitors. *Nat. Commun.* 6:8518. 10.1038/ncomms9518 PMC4600759 26439639

[B86] Gergely P. Nuesslein-Hildesheim B. Guerini D. Brinkmann V. Traebert M. Bruns C. (2012). The selective sphingosine 1-phosphate receptor modulator BAF312 redirects lymphocyte distribution and has species-specific effects on heart rate. *Br. J. Pharmacol.* 167 1035–1047. 10.1111/j.1476-5381.2012.02061.x 22646698 PMC3485666

[B87] Gessi S. Varani K. Merighi S. Cattabriga E. Pancaldi C. Szabadkai Y. (2005). Expression, pharmacological profile, and functional coupling of A(2B) receptors in a recombinant system and in peripheral blood cells using a novel selective antagonist radioligand, H-3 MRE 2029-F20. *Mol. Pharmacol.* 67 2137–2147. 10.1124/mol.104.009225 15788741

[B88] Gessiaa S. Merighi S. Varani K. Leung E. Mac Lennan S. Borea P. A. (2008). The A(3) adenosine receptor: an enigmatic player in cell biology. *Pharmacol. Ther.* 117 123–140. 10.1016/j.pharmthera.2007.09.002 18029023

[B89] Goncalves F. Q. Pires J. Pliassova A. Beleza R. Lemos C. Marques J. M. (2015). Adenosine A(2b) receptors control A(1) receptor-mediated inhibition of synaptic transmission in the mouse hippocampus. *Eur. J. Neurosci.* 41 876–886. 10.1111/ejn.12851 25704806

[B90] Goncalves M. L. Ribeiro J. A. (1996). Adenosine A(2) receptor activation facilitates Ca-45(2+) uptake by rat brain synaptosomes. *Eur. J. Pharmacol.* 310 257–261. 10.1016/0014-2999(96)00383-4 8884224

[B91] Gonzalez-Fernandez E. Sanchez-Gomez M. V. Perez-Samartin A. Arellano R. O. Matute C. (2014). A(3) Adenosine receptors mediate oligodendrocyte death and ischemic damage to optic nerve. *Glia* 62 199–216. 10.1002/glia.22599 24311446

[B92] Graeler M. Goetzl E. J. (2002). Activation-regulated expression and chemotactic function of sphingosine 1-phosphate receptors in mouse splenic T cells. *FASEB J.* 16 1874–1878. 10.1096/fj.02-0548com 12468451

[B93] Graler M. H. Goetzl E. J. (2004). The immunosuppressant FTY720 down-regulates sphingosine 1-phosphate G protein-coupled receptors. *FASEB J.* 18 551–553. 10.1096/fj.03-0910fje 14715694

[B94] Grinspan J. (2002). Cells and signaling in oligodendrocyte development. *J. Neuropathol. Exp. Neurol.* 61 297–306. 10.1093/jnen/61.4.297 11939585

[B95] Gutman G. A. Chandy K. G. Grissmer S. Lazdunski M. McKinnon D. Pardo L. A. (2005). International Union of Pharmacology. LIII. Nomenclature and molecular relationships of voltage-gated potassium channels. *Pharmacol. Rev.* 57 473–508. 10.1124/pr.57.4.10 16382104

[B96] Hait N. C. Allegood J. Maceyka M. Strub G. M. Harikumar K. B. Singh S. K. (2009). Regulation of Histone acetylation in the nucleus by sphingosine-1-phosphate. *Science* 325 1254–1257. 10.1126/science.1176709 19729656 PMC2850596

[B97] Hait N. C. Sarkar S. Le Stunff H. Mikami A. Maceyka M. Milstien S. (2005). Role of sphingosine kinase 2 in cell migration toward epidermal growth factor. *J. Biol. Chem.* 280 29462–29469. 10.1074/jbc.m502922200 15951439

[B98] Hajny S. Christoffersen C. (2017). A novel perspective on the ApoM-S1P Axis, highlighting the metabolism of ApoM and its role in liver fibrosis and neuroinflammation. *Int. J. Mol. Sci.* 18:1636. 10.3390/ijms18081636 28749426 PMC5578026

[B99] Hanada K. (2003). Serine palmitoyltransferase, a key enzyme of sphingolipid metabolism. *Bioch. Biophys. Acta.* 1632 16–30. 10.1016/s1388-1981(03)00059-3 12782147

[B100] Hannun Y. A. Obeid L. M. (2008). Principles of bioactive lipid signalling: lessons from sphingolipids. *Nat. Rev. Mol. Cell Biol.* 9 139–150. 10.1038/nrm2329 18216770

[B101] Hasko G. Linden J. Cronstein B. Pacher P. (2008). Adenosine receptors: therapeutic aspects for inflammatory and immune diseases. *Nat. Rev. Drug Discov.* 7 759–770. 18758473 10.1038/nrd2638 PMC2568887

[B102] Hisano Y. Kobayashi N. Yamaguchi A. Nishi T. (2012). Mouse SPNS2 functions as a sphingosine-1-phosphate transporter in vascular endothelial cells. *Plos One* 7:e38941. 10.1371/journal.pone.0038941 22723910 PMC3379171

[B103] Hla T. Venkataraman K. Michaud J. (2008). The vascular S1P gradient - cellular sources and biological significance. *Biochim. Biophys. Acta* 1781 477–482. 10.1016/j.bbalip.2008.07.003 18674637 PMC2636563

[B104] Hou H. Q. Cao R. J. Miao J. Sun Y. F. Liu X. Q. Song X. J. (2016). Fingolimod ameliorates the development of experimental autoimmune encephalomyelitis by inhibiting Akt-mTOR axis in mice. *Int. Immunopharmacol.* 30 171–178. 10.1016/j.intimp.2015.11.024 26632437

[B105] Hu Y. H. Lee X. H. Ji B. X. Guckian K. Apicco D. Pepinsky R. B. (2011). Sphingosine 1-phosphate receptor modulator fingolimod (FTY720) does not promote remyelination in vivo. *Mol. Cell. Neurosci.* 48 72–81. 10.1016/j.mcn.2011.06.007 21740973

[B106] Igarashi N. Okada T. Hayashi S. Fujita T. Jahangeer S. Nakamura S. (2003). Sphingosine kinase 2 is a nuclear protein and inhibits DNA synthesis. *J. Biol. Chem.* 278 46832–46839. 10.1074/jbc.m306577200 12954646

[B107] Im D. S. Heise C. E. Ancellin N. O’Dowd B. F. Shei G. J. Heavens R. P. (2000). Characterization of a novel sphingosine 1-phosphate receptor, Edg-8. *J. Biol. Chem.* 275 14281–14286. 10.1074/jbc.275.19.14281 10799507

[B108] Imamura T. Ohgane J. Ito S. Ogawa T. Hattori N. Tanaka S. (2001). CpG island of rat sphingosine kinase-1 gene: tissue-dependent DNA methylation status and multiple alternative first exons. *Genomics* 76 117–125. 10.1006/geno.2001.6607 11560121

[B109] Ingwersen J. Wingerath B. Graf J. Lepka K. Hofrichter M. Schröter F. (2016). Dual roles of the adenosine A2a receptor in autoimmune neuroinflammation. *J. Neuroinflam.* 13:48. 10.1186/s12974-016-0512-z PMC4768407 26920550

[B110] Inoue K. (2017). Molecular basis of nucleobase transport systems in mammals. *Biol. Pharm. Bull.* 40 1130–1138. 10.1248/bpb.b17-00374 28768993

[B111] Ishii I. Fukushima N. Ye X. Q. Chun J. (2004). Lysophospholipid receptors: signaling and biology. *Annu. Rev. Biochem.* 73 321–354. 15189145 10.1146/annurev.biochem.73.011303.073731

[B112] Jacobson K. A. Gao Z. G. (2006). Adenosine receptors as therapeutic targets. *Nat. Rev. Drug Discov.* 5 247–264. 16518376 10.1038/nrd1983 PMC3463109

[B113] Jaillard C. Harrison S. Stankoff B. Aigrot M. S. Calver A. R. Duddy G. (2005). Edg8/S1P5: an oligodendroglial receptor with dual function on process retraction and cell survival. *J. Neurosci.* 25 1459–1469. 10.1523/jneurosci.4645-04.2005 15703400 PMC6726002

[B114] Johnson K. R. Becker K. P. Facchinetti M. M. Hannun Y. A. Obeid L. M. (2002). PKC-dependent activation of sphingosine kinase 1 and translocation to the plasma membrane - Extracellular release of sphingosine-1-phosphate induced by phorbol 12-myristate 13-acetate (PMA). *J. Biol. Chem.* 277 35257–35262. 12124383 10.1074/jbc.M203033200

[B115] Johnston J. B. Silva C. Gonzalez G. Holden J. Warren K. G. Metz L. M. (2001). Diminished adenosine A1 receptor expression on macrophages in brain and blood of patients with multiple sclerosis. *Ann. Neurol.* 49 650–658. 10.1002/ana.1007 11357956

[B116] Jung C. G. Kim H. J. Miron V. E. Cook S. Kennedy T. E. Foster C. A. (2007). Functional consequences of S1P receptor modulation in rat oligodendroglial lineage cells. *Glia* 55 1656–1667. 10.1002/glia.20576 17876806

[B117] Jung M. Sommer I. Schachner M. Nave K. A. (1996). Monoclonal antibody O10 defines a conformationally sensitive cell-surface epitope of proteolipid protein (PLP): evidence that PLP misfolding underlies dysmyelination in mutant mice. *J. Neurosci.* 16 7920–7929. 10.1523/jneurosci.16-24-07920.1996 8987820 PMC6579218

[B118] Kappos L. Radue E. W. O’Connor P. Polman C. Hohlfeld R. Calabresi P. (2010). A Placebo-controlled trial of oral fingolimod in relapsing multiple sclerosis. *N. Engl. J. Med.* 362 387–401. 20089952 10.1056/NEJMoa0909494

[B119] Karadottir R. Hamilton N. B. Bakiri Y. Attwell D. (2008). Spiking and nonspiking classes of oligodendrocyte precursor glia in CNS white matter. *Nat. Neurosci.* 11 450–456. 10.1038/nn2060 18311136 PMC2615224

[B120] Kataoka H. Sugahara K. Shimano K. Teshima K. Koyama M. Fukunari A. (2005). FTY720, Sphingosine 1-phosphate receptor modulator, ameliorates experimental autoimmune encephalomyelitis by inhibition of T cell infiltration. *Cell. Mol. Immunol.* 2 439–448. 16426494

[B121] Kettenmann H. Blankenfeld G. V. Trotter J. (1991). Physiological properties of oligodendrocytes during development. *Ann. N. Y. Acad. Sci.* 633 64–77. 10.1111/j.1749-6632.1991.tb15596.x 1724138

[B122] Kharel Y. Lee S. Snyder A. H. Heasley-O’neill S. L. Morris M. A. Setiady Y. (2005). Sphingosine kinase 2 is required for modulation of lymphocyte traffic by FTY720. *J. Biol. Chem.* 280 36865–36872. 10.1074/jbc.m506293200 16093248

[B123] Kim H. J. Miron V. E. Dukala D. Proia R. L. Ludwin S. K. Traka M. (2011). Neurobiological effects of sphingosine 1-phosphate receptor modulation in the cuprizone model. *FASEB J.* 25 1509–1518. 10.1096/fj.10-173203 21248243 PMC3079302

[B124] Kim S. Bielawski J. Yang H. Kong Y. Zhou B. Y. Li J. R. (2018). Functional antagonism of sphingosine-1-phosphate receptor 1 prevents cuprizone-induced demyelination. *Glia* 66 654–669. 10.1002/glia.23272 29193293 PMC5773114

[B125] Knutson P. Ghiani C. A. Zhou J. M. Gallo V. McBain C. J. (1997). K+ channel expression and cell proliferation are regulated by intracellular sodium and membrane depolarization in oligodendrocyte progenitor cells. *J. Neurosci.* 17 2669–2682. 10.1523/jneurosci.17-08-02669.1997 9092588 PMC6573116

[B126] Kobayashi N. Kawasaki-Nishi S. Otsuka M. Hisano Y. Yamaguchi A. Nishi T. (2018). MFSD2B is a sphingosine 1-phosphate transporter in erythroid cells. *Sci. Rep.* 8:4969. 10.1038/s41598-018-23300-x PMC5862976 29563527

[B127] Kohno M. Momoi M. Oo M. L. Paik J. H. Lee Y. M. Venkataraman K. (2006). Intracellular role for sphingosine kinase 1 in intestinal adenoma cell proliferation. *Mol. Cell. Biol.* 26 7211–7223. 10.1128/mcb.02341-05 16980623 PMC1592880

[B128] Koscso B. Csoka B. Pacher P. Hasko G. (2011). Investigational A(3) adenosine receptor targeting agents. *Expert Opin. Investig. Drugs* 20 757–768. 10.1517/13543784.2011.573785 21457061 PMC3613226

[B129] Ksiazek M. Chacinska M. Chabowski A. Baranowski M. (2015). Sources, metabolism, and regulation of circulating sphingosine-1-phosphate. *J. Lipid Res.* 56 1271–1281. 10.1194/jlr.r059543 26014962 PMC4479332

[B130] Latini S. Bordoni F. Pedata F. Corradetti R. (1999). Extracellular adenosine concentrations during in vitro ischaemia in rat hippocampal slices. *Br. J. Pharmacol.* 127 729–739. 10.1038/sj.bjp.0702591 10401564 PMC1566061

[B131] Latini S. Pedata F. (2001). Adenosine in the central nervous system: release mechanisms and extracellular concentrations. *J. Neurochem.* 79 463–484. 10.1046/j.1471-4159.2001.00607.x 11701750

[B132] Lecca D. Trincavelli M. L. Gelosa P. Sironi L. Ciana P. Fumagalli M. (2008). The recently identified P2Y-Like receptor GPR17 is a sensor of brain damage and a new target for brain repair. *PLoS One* 3:e3579. 10.1371/journal.pone.0003579 18974869 PMC2570486

[B133] Levi G. Gallo V. Ciotti M. T. (1986). Bipotential precursors of putative fibrous astrocytes and oligodendrocytes in rat cerebellar cultures express distinct surface-features and neuron-like gamma-aminobutyric-acid transport. *Proc. Natl. Acad. Sci. U.S.A.* 83 1504–1508. 10.1073/pnas.83.5.1504 3513179 PMC323105

[B134] Levine J. M. Reynolds R. Fawcett J. W. (2001). The oligodendrocyte precursor cell in health and disease. *Trends Neurosci.* 24 39–47. 10.1016/s0166-2236(00)01691-x 11163886

[B135] Ligon K. L. Kesari S. Kitada M. Sun T. Arnett H. A. Alberta J. A. (2006). Development of NG2 neural progenitor cells requires Olig gene function. *Proc. Natl. Acad. Sci. U.S.A.* 103 7853–7858. 10.1073/pnas.0511001103 16682644 PMC1472534

[B136] Liu G. P. Zhang W. Guo J. Kong F. Q. Zhou S. M. Chen S. (2018). Adenosine binds predominantly to adenosine receptor A1 subtype in astrocytes and mediates an immunosuppressive effect. *Brain Res.* 1700 47–55. 10.1016/j.brainres.2018.06.021 29935155

[B137] Liu Y. Alahiri M. Ulloa B. Xie B. X. Sadiq S. A. (2018). Adenosine A2A receptor agonist ameliorates EAE and correlates with Th1 cytokine-induced blood brain barrier dysfunction via suppression of MLCK signaling pathway. *Immun. Inflamm. Dis.* 6 72–80. 10.1002/iid3.187 29027376 PMC5818446

[B138] Liu H. Chakravarty D. Maceyka M. Milstien S. Spiegel S. (2002). Sphingosine kinases: a novel family of lipid kinases. *Prog. Nucleic Acid Res. Mol. Biol.* 71 493–511. 10.1016/s0079-6603(02)71049-0 12102559

[B139] Liu H. Toman R. E. Goparaju S. K. Maceyka M. Nava V. E. Sankala H. (2003). Sphingosine kinase type 2 is a putative BH3-only protein that induces apoptosis. *J. Biol. Chem.* 278 40330–40336. 10.1074/jbc.m304455200 12835323

[B140] Liu H. Zhang Y. J. Wu H. Y. D’Alessandro A. Yegutkin G. G. Song A. (2016). Beneficial role of erythrocyte adenosine A2B receptor-mediated AMP-activated protein kinase activation in high-altitude hypoxia. *Circulation* 134 405–421. 10.1161/circulationaha.116.021311 27482003 PMC6168195

[B141] Liu Y. Q. Ma Y. Y. Du B. Y. Wang Y. T. Yang G. Y. Bi X. Y. (2020). Mesenchymal stem cells attenuated blood-brain barrier disruption via downregulation of aquaporin-4 expression in EAE mice. *Mol. Neurobiol.* 57 3891–3901. 10.1007/s12035-020-01998-z 32613467 PMC7399688

[B142] Lopes L. V. Cunha R. A. Kull B. Fredholm B. B. Ribeiro J. A. (2002). Adenosine A(2A) receptor facilitation of hippocampal synaptic transmission is dependent on tonic A(1) receptor inhibition. *Neuroscience* 112 319–329. 10.1016/s0306-4522(02)00080-5 12044450

[B143] Lundgaard I. Luzhynskaya A. Stockley J. H. Wang Z. Evans K. A. Swire M. (2013). Neuregulin and BDNF induce a switch to NMDA receptor-dependent Myelination by Oligodendrocytes. *Plos Biol.* 11:e1001743. 10.1371/journal.pbio.1001743 24391468 PMC3876980

[B144] Maceyka M. Harikumar K. B. Milstien S. Spiegel S. (2012). Sphingosine-1-phosphate signaling and its role in disease. *Tren. Cell Biol.* 22 50–60. 10.1016/j.tcb.2011.09.003 22001186 PMC3253987

[B145] Maceyka M. Sankala H. Hait N. C. Le Stunff H. Liu H. Toman R. (2005). SphK1 and SphK2, sphingosine kinase isoenzymes with opposing functions in sphingolipid metabolism. *J. Biol. Chem.* 280 37118–37129. 10.1074/jbc.m502207200 16118219

[B146] Malerba F. Paoletti F. Ercole B. B. Materazzi S. Nassini R. Coppi E. (2015). Functional characterization of human ProNGF and NGF mutants: identification of NGF P61SR100E as a “Painless” lead investigational candidate for therapeutic applications. *PLoS One* 10:e0136425. 10.1371/journal.pone.0136425 26371475 PMC4570711

[B147] Manalo J. M. Liu H. Ding D. L. Hicks J. Sun H. Salvi R. (2020). Adenosine A2B receptor: a pathogenic factor and a therapeutic target for sensorineural hearing loss. *FASEB J*. 34 15771–15787. 10.1096/fj.202000939r 33131093

[B148] Mandala S. Hajdu R. Bergstrom J. Quackenbush E. Xie J. Milligan J. (2002). Alteration of lymphocyte trafficking by sphingosine-1-phosphate receptor agonists. *Science* 296 346–349. 10.1126/science.1070238 11923495

[B149] Maraula G. Lana D. Coppi E. Gentile F. Mello T. Melani A. (2014). The selective antagonism of P2X7 and P2Y1 receptors prevents synaptic failure and affects cell proliferation induced by oxygen and glucose deprivation in rat dentate gyrus. *PLoS One* 9:e115273. 10.1371/journal.pone.0115273 25526634 PMC4272279

[B150] Maraula G. Traini C. Mello T. Coppi E. Galli A. Pedata F. (2013). Effects of oxygen and glucose deprivation on synaptic transmission in rat dentate gyrus: role of A(2A) adenosine receptors. *Neuropharmacology* 67 511–520. 10.1016/j.neuropharm.2012.12.002 23261865

[B151] Matloubian M. Lo C. G. Cinamon G. Lesneski M. J. Xu Y. Brinkmann V. (2004). Lymphocyte egress from thymus and peripheral lymphoid organs is dependent on S1P receptor 1. *Nature* 427 355–360. 10.1038/nature02284 14737169

[B152] Mayne M. Shepel P. N. Jiang Y. Geiger J. D. Power C. (1999). Dysregulation of adenosine A(1) receptor-mediated cytokine expression in peripheral blood mononuclear cells from multiple sclerosis patients. *Ann. Neurol.* 45 633–639. 10.1002/1531-8249(199905)45:5<633::aid-ana12>3.0.co;2-x 10319886

[B153] Mehling M. Brinkmann V. Antel J. Bar-Or A. Goebels N. Vedrine C. (2008). FTY720 therapy exerts differential effects on T cell subsets in multiple sclerosis. *Neurology* 71 1261–1267. 10.1212/01.wnl.0000327609.57688.ea 18852441

[B154] Melani A. Cipriani S. Vannucchi M. G. Nosi D. Donati C. Bruni P. (2009). Selective adenosine A(2a) receptor antagonism reduces JNK activation in oligodendrocytes after cerebral ischaemia. *Brain* 132 1480–1495. 10.1093/brain/awp076 19359287

[B155] Michaud J. Kohno M. Proia R. L. Hla T. (2006). Normal acute and chronic inflammatory responses in sphingosine kinase 1 knockout mice. *FEBS Lett.* 580 4607–4612. 10.1016/j.febslet.2006.07.035 16876794

[B156] Mills J. H. Alabanza L. M. Mahamed D. A. Bynoe M. S. (2012). Extracellular adenosine signaling induces CX3CL1 expression in the brain to promote experimental autoimmune encephalomyelitis. *J. Neuroinflamm.* 9:193. 10.1186/1742-2094-9-193 PMC3458968 22883932

[B157] Miron V. E. Hall J. A. Kennedy T. E. Soliven B. Antel J. P. (2008a). Cyclical and dose-dependent responses of adult human mature oligodendrocytes to fingolimod. *Am. J. Pathol.* 173 1143–1152. 10.2353/ajpath.2008.080478 18772343 PMC2543081

[B158] Miron V. E. Jung C. G. Kim H. J. Kennedy T. E. Soliven B. Antel J. P. (2008b). FTY720 modulates human oligodendrocyte progenitor process extension and survival. *Ann. Neurol.* 63 61–71. 10.1002/ana.21227 17918267

[B159] Miron V. E. Schubart A. Antel J. P. (2008c). Central nervous system-directed effects of FTY720 (fingolimod). *J. Neurol. Sci.* 274 13–17. 10.1016/j.jns.2008.06.031 18678377

[B160] Miron V. E. Ludwin S. K. Darlington P. J. Jarjour A. A. Soliven B. Kennedy T. E. (2010). Fingolimod (FTY720) enhances remyelination following demyelination of organotypic cerebellar slices. *Am. J. Pathol.* 176 2682–2694. 10.2353/ajpath.2010.091234 20413685 PMC2877831

[B161] Mitra P. Oskeritzian C. A. Payne S. G. Beaven M. A. Milstien S. Spiegel S. (2006). Role of ABCO in export of sphingosine-1-phosphate from mast cells. *Proc. Natl. Acad. Sci. U.S.A.* 103 16394–16399. 10.1073/pnas.0603734103 17050692 PMC1637593

[B162] Mizugishi K. Yamashita T. Olivera A. Miller G. F. Spiegel S. Proia R. L. (2005). Essential role for sphingosine kinases in neural and vascular development. *Mol. Cell. Biol.* 25 11113–11121. 10.1128/mcb.25.24.11113-11121.2005 16314531 PMC1316977

[B163] Montesinos M. C. Yap J. S. Desai A. Posadas I. McCrary C. T. Cronstein B. N. (2000). Reversal of the antiinflammatory effects of methotrexate by the nonselective adenosine receptor antagonists theophylline and caffeine - Evidence that the antiinflammatory effects of methotrexate are mediated via multiple adenosine receptors in rat adjuvant arthritis. *Arthrit. Rheum.* 43 656–663. 10.1002/1529-0131(200003)43:3<656::aid-anr23>3.0.co;2-h 10728760

[B164] Mutoh T. Rivera R. Chun J. (2012). Insights into the pharmacological relevance of lysophospholipid receptors. *Br. J. Pharmacol.* 165 829–844. 10.1111/j.1476-5381.2011.01622.x 21838759 PMC3312481

[B165] Muzzi M. Coppi E. Pugliese A. M. Chiarugi A. (2013). Anticonvulsant effect of AMP by direct activation of adenosine A1 receptor. *Exp. Neurol.* 250 189–193. 10.1016/j.expneurol.2013.09.010 24056265

[B166] Nave K. A. (2010). Myelination and support of axonal integrity by glia. *Nature* 468 244–252. 10.1038/nature09614 21068833

[B167] Neubauer H. A. Pham D. H. Zebol J. R. Moretti P. A. Peterson A. L. Leclercq T. M. (2016). An oncogenic role for sphingosine kinase 2. *Oncotarget* 7 64886–64899. 27588496 10.18632/oncotarget.11714 PMC5323123

[B168] Neumann B. Segel M. Chalut K. J. Franklin R. J. M. (2019). Remyelination and ageing: Reversing the ravages of time. *Mult. Scler. J.* 25 1835–1841. 10.1177/1352458519884006 31687878 PMC7682531

[B169] Novgorodov A. S. El-Alwani M. Bielawski J. Obeid L. M. Gudz T. I. (2007). Activation of sphingosine-1-phosphate receptor S1P5 inhibits oligodendrocyte progenitor migration. *FASEB J.* 21 1503–1514. 10.1096/fj.06-7420com 17255471

[B170] Nystad A. E. Lereim R. R. Wergeland S. Oveland E. Myhr K. M. Bo L. (2020). Fingolimod downregulates brain sphingosine-1-phosphate receptor 1 levels but does not promote remyelination or neuroprotection in the cuprizone model. *J. Neuroimmunol.* 339:577091. 10.1016/j.jneuroim.2019.577091 31739156

[B171] Obinata H. Kuo A. Wada Y. Swendeman S. Liu C. H. Blaho V. A. (2019). Identification of ApoA4 as a sphingosine 1-phosphate chaperone in ApoM- and albumin-deficient mice S. *J. Lipid Res.* 60 1912–1921. 10.1194/jlr.ra119000277 31462513 PMC6824498

[B172] Okada T. Ding G. Sonoda H. Kajimoto T. Haga Y. Khosrowbeygi A. (2005). Involvement of N-terminal-extended form of sphingosine kinase 2 in serum-dependent regulation of cell proliferation and apoptosis. *J. Biol. Chem.* 280 36318–36325. 10.1074/jbc.m504507200 16103110

[B173] Okada T. Kajimoto T. Jahangeer S. Nakamura S. (2009). Sphingosine kinase/sphingosine 1-phosphate signalling in central nervous system. *Cell. Signal.* 21 7–13. 10.1016/j.cellsig.2008.07.011 18694820

[B174] Olivera A. Spiegel S. (2001). Sphingosine kinase: a mediator of vital cellular functions. *Prostaglandins Other Lipid Mediat.* 64 123–134. 10.1016/s0090-6980(01)00108-3 11324702

[B175] Othman T. Yan H. L. Rrvkees S. A. (2003). Oligodendrocytes express functional A1 adenosine receptors that stimulate cellular migration. *Glia* 44 166–172. 10.1002/glia.10281 14515332

[B176] Papadopoulos D. Rundle J. Patel R. Marshall I. Stretton J. Eaton R. (2010). FTY720 Ameliorates MOG-induced experimental autoimmune encephalomyelitis by suppressing both cellular and humoral immune responses. *J. Neurosci. Res.* 88 346–359. 10.1002/jnr.22196 19658199

[B177] Pappu R. Schwab S. R. Cornelissen I. Pereira J. P. Regard J. B. Xu Y. (2007). Promotion of lymphocyte egress into blood and lymph by distinct sources of sphingosine-1-phosphate. *Science* 316 295–298. 10.1126/science.1139221 17363629

[B178] Patel J. R. Klein R. S. (2011). Mediators of oligodendrocyte differentiation during remyelination. *FEBS Lett.* 585 3730–3737. 10.1016/j.febslet.2011.04.037 21539842 PMC3158966

[B179] Paugh S. W. Payne S. G. Barbour S. E. Milstien S. Spiegel S. (2003). The immunosuppressant FTY720 is phosphorylated by sphingosine kinase type 2. *FEBS Lett.* 554 189–193. 10.1016/s0014-5793(03)01168-2 14596938

[B180] Pedata F. Melani A. Pugliese A. M. Coppi E. Cipriani S. Traini C. (2007). The role of ATP and adenosine in the brain under normoxic and ischemic conditions. *Purinergic Signal.* 3 299–310. 10.1007/s11302-007-9085-8 18404443 PMC2072927

[B181] Pedata F. Pugliese A. M. Coppi E. Dettori I. Maraula G. Cellai L. (2014). Adenosine A(2A) receptors modulate acute injury and neuroinflammation in brain ischemia. *Med. Inflamm*. 2014:805198. 10.1155/2014/805198 PMC4138795 25165414

[B182] Peterfreund R. A. MacCollin M. Gusella J. Fink J. S. (1996). Characterization and expression of the human A2a adenosine receptor gene. *J. Neurochem.* 66 362–368. 10.1046/j.1471-4159.1996.66010362.x 8522976

[B183] Pitson S. M. Moretti P. A. B. Zebol J. R. Xia P. Gamble J. R. Vadas M. A. (2000). Expression of a catalytically inactive sphingosine kinase mutant blocks agonist-induced sphingosine kinase activation - A dominant-negative sphingosine kinase. *J. Biol. Chem.* 275 33945–33950. 10.1074/jbc.m006176200 10944534

[B184] Popoli P. Pepponi R. (2012). Potential therapeutic relevance of adenosine A(2B) and A(2A) receptors in the central nervous system. *Cns Neurol. Disord. Drug Targets* 11 664–674. 10.2174/187152712803581100 22963436

[B185] Pringle N. P. Mudhar H. S. Collarini E. J. Richardson W. D. (1992). Pdgf receptors in the rat cns - during late neurogenesis, pdgf alpha-receptor expression appears to be restricted to glial-cells of the oligodendrocyte lineage. *Development* 115 535–551. 10.1242/dev.115.2.535 1425339

[B186] Proia R. L. Hla T. (2015). Emerging biology of sphingosine-1-phosphate: its role in pathogenesis and therapy. *J. Clin. Invest.* 125 1379–1387. 10.1172/jci76369 25831442 PMC4409021

[B187] Pyne S. Adams D. R. Pyne N. J. (2016). Sphingosine 1-phosphate and sphingosine kinases in health and disease: recent advances. *Prog. Lipid Res.* 62 93–106. 10.1016/j.plipres.2016.03.001 26970273

[B188] Qin J. D. Berdyshev E. Goya J. Natarajan V. Dawson G. (2010). Neurons and oligodendrocytes recycle Sphingosine 1-phosphate to ceramide significance for apoptosis and multiple sclerosis. *J. Biol. Chem.* 285 14134–14143. 10.1074/jbc.m109.076810 20215115 PMC2863199

[B189] Rajasundaram S. (2018). Adenosine A2A receptor signaling in the immunopathogenesis of experimental autoimmune encephalomyelitis. *Front. Immunol.* 9:402. 10.3389/fimmu.2018.00402 29559972 PMC5845642

[B190] Ramkumar V. Stiles G. L. Beaven M. A. Ali H. (1993). The a(3) adenosine receptor is the unique adenosine receptor which facilitates release of allergic mediators in mast-cells. *J. Biol. Chem.* 268 16887–16890. 10.1016/s0021-9258(19)85277-8 8349579

[B191] Rissanen E. Virta J. R. Paavilainen T. Tuisku J. Helin S. Luoto P. (2013). Adenosine A2A receptors in secondary progressive multiple sclerosis: a C-11 TMSX brain PET study. *J. Cereb. Blood Flow Metab.* 33 1394–1401. 10.1038/jcbfm.2013.85 23695433 PMC3764386

[B192] Romanelli E. Merkler D. Mezydlo A. Weil M. T. Weber M. S. Nikić I. (2016). Myelinosome formation represents an early stage of oligodendrocyte damage in multiple sclerosis and its animal model. *Nat. Commun.* 7:13275. 10.1038/ncomms13275 PMC5116090 27848954

[B193] Saab A. S. Tzvetavona I. D. Trevisiol A. Baltan S. Dibaj P. Kusch K. (2016). Oligodendroglial NMDA receptors regulate glucose import and axonal energy metabolism. *Neuron* 91 119–132. 10.1016/j.neuron.2016.05.016 27292539 PMC9084537

[B194] Saini H. S. Coelho R. P. Goparaju S. K. Jolly P. S. Maceyka M. Spiegel S. (2005). Novel role of sphingosine kinase 1 as a mediator of neurotrophin-3 action in oligodendrocyte progenitors. *J. Neurochem.* 95 1298–1310. 10.1111/j.1471-4159.2005.03451.x 16313513

[B195] Sarkar S. Maceyka M. Hait N. C. Paugh S. W. Sankala H. Milstien S. (2005). Sphingosine kinase 1 is required for migration, proliferation and survival of MCF-7 human breast cancer cells. *FEBS Lett.* 579 5313–5317. 10.1016/j.febslet.2005.08.055 16194537

[B196] Schwab S. R. Pereira J. P. Matloubian M. Xu Y. Huang Y. Cyster J. G. (2005). Lymphocyte sequestration through S1P lyase inhibition and disruption of S1P gradients. *Science* 309 1735–1739. 10.1126/science.1113640 16151014

[B197] Scolding N. J. Frith S. Linington C. Morgan B. P. Campbell A. K. Compston D. A. S. (1989). Myelin-oligodendrocyte glycoprotein (mog) is a surface marker of oligodendrocyte maturation. *J. Neuroimmunol.* 22 169–176. 10.1016/0165-5728(89)90014-3 2649509

[B198] Shrager P. Novakovic S. D. (1995). Control of myelination, axonal growth, and synapse formation in spinal-cord explants by ion channels and electrical-activity. *Dev. Brain res.* 88 68–78. 10.1016/0165-3806(95)00081-n 7493408

[B199] Simon C. Gotz M. Dimou L. (2011). Progenitors in the adult cerebral cortex: cell cycle properties and regulation by physiological stimuli and injury. *Glia* 59 869–881. 10.1002/glia.21156 21446038

[B200] Slowik A. Schmidt T. Beyer C. Amor S. Clarner T. Kipp M. (2015). The sphingosine 1-phosphate receptor agonist FTY720 is neuroprotective after cuprizone-induced CNS demyelination. *Br. J. Pharmacol.* 172 80–92. 10.1111/bph.12938 25220526 PMC4280969

[B201] Smith P. A. Schmid C. Zurbruegg S. Jivkov M. Doelemeyer A. Theil D. (2018). Fingolimod inhibits brain atrophy and promotes brain-derived neurotrophic factor in an animal model of multiple sclerosis. *J. Neuroimmunol.* 318 103–113. 10.1016/j.jneuroim.2018.02.016 29530550

[B202] Soliven B. Miron V. Chun J. (2011). The neurobiology of sphingosine 1-phosphate signaling and sphingosine 1-phosphate receptor modulators. *Neurology* 76 S9–S14. 21339490 10.1212/WNL.0b013e31820d9507

[B203] Soliven B. Szuchet S. Arnason B. G. W. Nelson D. J. (1988). Forskolin and phorbol esters decrease the same k+ conductance in cultured oligodendrocytes. *J. Memb. Biol.* 105 177–186. 10.1007/bf02009170 3216367

[B204] Sontheimer H. Kettenmann H. (1988). Heterogeneity of potassium currents in cultured oligodendrocytes. *Glia* 1 415–420. 10.1002/glia.440010609 2976401

[B205] Sontheimer H. Trotter J. Schachner M. Kettenmann H. (1989). Channel expression correlates with differentiation stage during the development of oligodendrocytes from their precursor cells in culture. *Neuron* 2 1135–1145. 10.1016/0896-6273(89)90180-3 2560386

[B206] Spiegel S. Maczis M. A. Maceyka M. Milstien S. (2019). New insights into functions of the sphingosine-1-phosphate transporter SPNS2. *J. Lipid Res.* 60 484–489. 10.1194/jlr.s091959 30655317 PMC6399492

[B207] Spiegel S. Milstien S. (2003). Sphingosine-1-phosphate: an enigmatic signalling lipid. *Nat. Rev. Mol. Cell Biol.* 4 397–407. 10.1038/nrm1103 12728273

[B208] Spitzer S. O. Sitnikov S. Kamen Y. Evans K. A. Kronenberg-Versteeg D. Dietmann S. (2019). Oligodendrocyte progenitor cells become regionally diverse and heterogeneous with age. *Neuron* 101:459. 10.1016/j.neuron.2018.12.020 30654924 PMC6372724

[B209] Stellwagen D. Malenka R. C. (2006). Synaptic scaling mediated by glial TNF-alpha. *Nature* 440 1054–1059. 10.1038/nature04671 16547515

[B210] Stevens B. Porta S. Haak L. L. Gallo V. Fields R. D. (2002). Adenosine: a neuron-glial transmitter promoting myelination in the CNS in response to action potentials. *Neuron* 36 855–868. 12467589 10.1016/s0896-6273(02)01067-x PMC1201407

[B211] Strub G. M. Maceyka M. Hait N. C. Milstien S. Spiegel S. (2010). Extracellular and intracellular actions of sphingosine-1-Phosphate. *Adv. Exp. Med. Biols.* 688 141–155. 10.1007/978-1-4419-6741-1_10 PMC2951632 20919652

[B212] Strub G. M. Paillard M. Liang J. Gomez L. Allegood J. C. Hait N. C. (2011). Sphingosine-1-phosphate produced by sphingosine kinase 2 in mitochondria interacts with prohibitin 2 to regulate complex IV assembly and respiration. *FASEB J.* 25 600–612. 10.1096/fj.10-167502 20959514 PMC3023391

[B213] Szuchet S. Nielsen J. A. Lovas G. Domowicz M. S. de Velasco J. M. Maric D. (2011). The genetic signature of perineuronal oligodendrocytes reveals their unique phenotype. *Eur. J. Neurosci.* 34 1906–1922. 10.1111/j.1460-9568.2011.07922.x 22132705 PMC4286392

[B214] Taha T. A. Kitatani K. El-Alwani M. Bielawski J. Hannun Y. A. Obeid L. M. (2005). Loss of sphingosine kinase-1 activates the intrinsic pathway of programmed cell death: modulation of sphingolipid levels and the induction of apoptosis. *FASEB J.* 19:482. 10.1096/fj.05-4412fje 16507765

[B215] Takasugi N. Sasaki T. Suzuki K. Osawa S. Isshiki H. Hori Y. (2011). BACE1 activity is modulated by cell-associated sphingosine-1-Phosphate. *J. Neurosci.* 31 6850–6857. 10.1523/jneurosci.6467-10.2011 21543615 PMC4534000

[B216] Tsutsui S. Schnermann J. Noorbakhsh F. Henry S. Yong V. W. Winston B. W. (2004). A1 adenosine receptor upregulation and activation attenuates neuroinflammation and demyelination in a model of multiple sclerosis. *J. Neurosci.* 24 1521–1529. 10.1523/jneurosci.4271-03.2004 14960625 PMC6730323

[B217] Van Brocklyn J. R. Jackson C. A. Pearl D. K. Kotur M. S. Snyder P. J. Prior T. W. (2005). Sphingosine kinase-1 expression correlates with poor survival of patients with glioblastoma multiforme: Roles of sphingosine kinase isoforms in growth of glioblastoma cell lines. *J. Neuropathol. Exp. Neurol.* 64 695–705. 10.1097/01.jnen.0000175329.59092.2c 16106218

[B218] van Doom R. Pinheiro M. A. L. Kooij G. Lakeman K. van het Hof B. van der Pol S. M. (2012). Sphingosine 1-phosphate receptor 5 mediates the immune quiescence of the human brain endothelial barrier. *J. Neuroinflamm.* 9:133. 10.1186/1742-2094-9-133 PMC3425155 22715976

[B219] Varani K. Padovan M. Vincenzi F. Targa M. Trotta F. Govoni M. (2011). A(2A) and A(3) adenosine receptor expression in rheumatoid arthritis: upregulation, inverse correlation with disease activity score and suppression of inflammatory cytokine and metalloproteinase release. *Arthrit. Res. Ther.* 13:R197. 10.1186/ar3527 PMC3334647 22146575

[B220] Verkhratsky A. Burnstock G. (2014). Biology of purinergic signalling: its ancient evolutionary roots, its omnipresence and its multiple functional significance. *Bioessays* 36 697–705. 10.1002/bies.201400024 24782352

[B221] Verkhratsky A. N. Trotter J. Kettenmann H. (1990). Cultured glial precursor cells from mouse cortex express 2 types of calcium currents. *Neurosci. Lett.* 112 194–198. 10.1016/0304-3940(90)90202-k 2163037

[B222] Vincenzi F. Corciulo C. Targa M. Merighi S. Gessi S. Casetta I. (2013). Multiple sclerosis lymphocytes upregulate A(2A) adenosine receptors that are antiinflammatory when stimulated. *Eur. J. Immunol.* 43 2206–2216. 10.1002/eji.201343314 23661562

[B223] Vincenzi F. Ravani A. Pasquini S. Merighi S. Gessi S. Romagnoli R. (2016). Positive allosteric modulation of A(1) adenosine receptors as a novel and promising therapeutic strategy for anxiety. *Neuropharmacology* 111 283–292. 10.1016/j.neuropharm.2016.09.015 27639989

[B224] Wakai A. Wang J. H. Winter D. C. Street J. T. O’Sullivan R. G. Redmond H. P. (2001). Adenosine inhibits neutrophil vascular endothelial growth factor release and transendothelial migration via A(2B) receptor activation. *Shock* 15 297–301. 10.1097/00024382-200115040-00008 11303729

[B225] Wang M. Luo G. H. Liu H. Zhang Y. P. Wang B. Di D. M. (2019). Apolipoprotein M induces inhibition of inflammatory responses via the S1PR1 and DHCR24 pathways. *Mol. Med. Rep.* 19 1272–1283. 30569161 10.3892/mmr.2018.9747

[B226] Wang T. Xi N. N. Chen Y. Shang X. F. Hu Q. Chen J. F. (2014). Chronic caffeine treatment protects against experimental autoimmune encephalomyelitis in mice: therapeutic window and receptor subtype mechanism. *Neuropharmacology* 86 203–211. 10.1016/j.neuropharm.2014.06.029 25019206

[B227] Warrington A. E. Barbarese E. Pfeiffer S. E. (1992). Stage specific, (o4+galc-) isolated oligodendrocyte progenitors produce mbp+myelin invivo. *Dev. Neurosci.* 14 93–97. 10.1159/000111652 1382941

[B228] Webb M. Tharn C. S. Lin F. F. Lariosa-Willingham K. Yu N. C. Hale J. (2004). Sphingosine 1-phosphate receptor agonists attenuate relapsing-remitting experimental autoimmune encephalitis in SJL mice. *J. Neuroimm.* 153 108–121. 10.1016/j.jneuroim.2004.04.015 15265669

[B229] Wei W. Du C. S. Lv J. Zhao G. X. Li Z. X. Wu Z. Y. (2013). Blocking A(2B) adenosine receptor alleviates pathogenesis of experimental autoimmune encephalomyelitis via inhibition of IL-6 production and Th17 differentiation. *J. Immunol.* 190 138–146. 10.4049/jimmunol.1103721 23225885 PMC3539684

[B230] Weigert A. Schiffmann S. Sekar D. Ley S. Menrad H. Werno C. (2009). Sphingosine kinase 2 deficient tumor xenografts show impaired growth and fail to polarize macrophages towards an anti-inflammatory phenotype. *Int. J. Cancer* 125 2114–2121. 10.1002/ijc.24594 19618460

[B231] Williamson A. V. Compston D. A. S. Randall A. D. (1997). Analysis of the ion channel complement of the rat oligodendrocyte progenitor in a commonly studied in vitro preparation. *Eur. J. Neurosci.* 9 706–720. 10.1111/j.1460-9568.1997.tb01419.x 9153577

[B232] Windh R. T. Lee M. J. Hla T. An S. Z. Barr A. J. Manning D. R. (1999). Differential coupling of the sphingosine 1-phosphate receptors Edg-1, Edg-3, and H218/Edg-5 to the G(i), G(q), and G(12) families of heterotrimeric G proteins. *J. Biol. Chem.* 274 27351–27358. 10.1074/jbc.274.39.27351 10488065

[B233] Yang D. Zhang Y. Nguyen H. G. Koupenova M. Chauhan A. K. Makitalo M. (2006). The A(2B) adenosine receptor protects against inflammation and excessive vascular adhesion. *J. Clin. Invest.* 116 1913–1923. 10.1172/jci27933 16823489 PMC1483170

[B234] Yao S. Q. Li Z. Z. Huang Q. Y. Li F. Wang Z. W. Augusto E. (2012). Genetic inactivation of the adenosine A2A receptor exacerbates brain damage in mice with experimental autoimmune encephalomyelitis. *J. Neurochem.* 123 100–112. 10.1111/j.1471-4159.2012.07807.x 22639925

[B235] Yazdi A. Baharvand H. Javan M. (2015). Enhanced remyelination following lysolecithin-induced demyelination in mice under treatment with fingolimod (FTY720). *Neuroscience* 311 34–44. 10.1016/j.neuroscience.2015.10.013 26475743

[B236] Yu L. Q. Frith M. C. Suzuki Y. Peterfreund R. A. Gearan T. Sugano S. (2004). Characterization of genomic organization of the adenosine A(2A) receptor gene by molecular and bioinformatics analyses. *Brain Res.* 1000 156–173. 10.1016/j.brainres.2003.11.072 15053963

[B237] Yu N. C. Lariosa-Willingham K. D. Lin F. F. Webb M. Rao T. S. (2004). Characterization of lysophosphatidic acid and sphingosine-1-phosphate-mediated signal transduction in rat cortical oligodendrocytes. *Glia* 45 17–27. 10.1002/glia.10297 14648542

[B238] Yu W. P. Collarini E. J. Pringle N. P. Richardson W. D. (1994). Embryonic expression of myelin genes - evidence for a focal source of oligodendrocyte precursors in the ventricular zone of the neurol tube. *Neuron.* 12 1353–1362. 10.1016/0896-6273(94)90450-2 7516688

[B239] Zemann B. Kinzel B. Muller M. Reuschel R. Mechtcheriakova D. Urtz N. (2006). Sphingosine kinase type 2 is essential for lymphopenia induced by the immunomodulatory drug FTY720. *Blood* 107 1454–1458. 10.1182/blood-2005-07-2628 16223773

[B240] Zhang J. Zhang Z. G. Li Y. Ding X. S. Shang X. Lu M. (2015). Fingolimod treatment promotes proliferation and differentiation of oligodendrocyte progenitor cells in mice with experimental autoimmune encephalomyelitis. *Neurobiol. Dis.* 76 57–66. 10.1016/j.nbd.2015.01.006 25680941

[B241] Zhang S. C. (2001). Defining glial cells during CNS development. *Nat. Rev. Neurosci.* 2 840–843. 10.1038/35097593 11715061

[B242] Zheng Z. Zeng Y. Z. Zhu X. Tan Y. Li Y. Li Q. (2019). ApoM-S1P modulates Ox-LDL-induced inflammation through the PI3K/Akt signaling pathway in HUVECs. *Inflammation* 42 606–617. 10.1007/s10753-018-0918-0 30377890

[B243] Zonouzi M. Renzi M. Farrant M. Cull-Candy S. G. (2011). Bidirectional plasticity of calcium-permeable AMPA receptors in oligodendrocyte lineage cells. *Nat. Neurosci.* 14 1430–U1103. 21983683 10.1038/nn.2942 PMC3204222

[B244] Zucker R. S. Regehr W. G. (2002). Short-term synaptic plasticity. *Annu. Rev. Physiol.* 64 355–405. 11826273 10.1146/annurev.physiol.64.092501.114547

